# Potential Future Impact of a Partially Effective HIV Vaccine in a Southern African Setting

**DOI:** 10.1371/journal.pone.0107214

**Published:** 2014-09-10

**Authors:** Andrew N. Phillips, Valentina Cambiano, Fumiyo Nakagawa, Deborah Ford, Jens D. Lundgren, Edith Roset-Bahmanyar, François Roman, Thierry Van Effelterre

**Affiliations:** 1 Research Department of Infection & Population Health, University College London, London, United Kingdom; 2 Institute of Clinical Trials and Methodology, University College London, London, United Kingdom; 3 CHIP, Department of Infectious Diseases, Rigshospitalet, University of Copenhagen, Copenhagen, Denmark; 4 GlaxoSmithKline Vaccines, Wavre, Belgium; CEA, France

## Abstract

**Background:**

It is important for public health and within the HIV vaccine development field to understand the potential population level impact of an HIV vaccine of partial efficacy—both in preventing infection and in reducing viral load in vaccinated individuals who become infected—in the context of a realistic future implementation scenario in resource limited settings.

**Methods:**

An individual level model of HIV transmission, progression and the effect of antiretroviral therapy was used to predict the outcome to 2060 of introduction in 2025 of a partially effective vaccine with various combinations of efficacy characteristics, in the context of continued ART roll-out in southern Africa.

**Results:**

In the context of our base case epidemic (in 2015 HIV prevalence 28% and incidence 1.7 per 100 person years), a vaccine with only 30% preventative efficacy could make a substantial difference in the rate with which HIV incidence declines; the impact on incidence in relative terms is projected to increase over time, with a projected 67% lower HIV incidence in 2060 compared with no vaccine introduction. The projected mean decline in the general adult population death rate 2040–2060 is 11%. A vaccine with no prevention efficacy but which reduces viral load by 1 log is predicted to result in a modest (14%) reduction in HIV incidence and an 8% reduction in death rate in the general adult population (mean 2040–2060). These effects were broadly similar in multivariable uncertainty analysis.

**Interpretation:**

Introduction of a partially effective preventive HIV vaccine would make a substantial long-term impact on HIV epidemics in southern Africa, in addition to the effects of ART. Development of an HIV vaccine, even of relatively low apparent efficacy at the individual level, remains a critical global public health goal.

## Introduction

There is evidence that incidence of HIV has declined in countries in southern Africa, likely due to population level awareness and changes in sexual risk behaviour, some effect of antiretroviral therapy in reducing infectivity, and other factors such as increased uptake of male circumcision [1]. However, prevalence remains high [1]. While continued roll out of ART will potentially lead to further reductions in incidence, and other effective prevention measures such as pre-exposure prophylaxis may be increasingly used, the need for additional approaches to help to limit new infections remains. The need for an HIV vaccine has been recognised from the start of the epidemic but progress has been limited and substantial challenges remain [2]. It is increasingly recognised that a vaccine with very high efficacy for preventing infection may not be attainable for the foreseeable future. Therefore it is relevant to consider what might be the effects of a vaccine with efficacy of perhaps as low as 30%, the estimated efficacy of the vaccine regimen used in the RV144 trial [3], and/or a vaccine that has limited or no efficacy in reducing risk of infection but which has efficacy in lowering viral load set point in people vaccinated (vaccinees) who become infected. Quantifying the population-level impact of such vaccines, particularly those that reduce viral load set point in those infected, is far from straightforward given the many inter-related factors that need to be accounted for and their highly dynamic nature. Mathematical models of HIV transmission are needed. In particular, modelling HIV at the individual-level is important, to account for the various heterogeneities between individuals and to evaluate the impact of a prophylactic HIV vaccine that can have effects on infection and on viral load in infected vaccinees. It is likely to be additionally informative to assess this impact within a realistic scenario which reflects current and potential future trends in the high prevalence region of southern Africa. The results of such modelling may guide vaccine development programmes, bringing clarity on the relative importance of the two aspects of efficacy and to understand the anticipated real life context into which a vaccine would be introduced.

In this paper we use an individual-based model [4], [5] to investigate the population-level impact of vaccination with a prophylactic HIV vaccine in a developing country setting with a generalised heterosexual epidemic (as in southern Africa). The model accounts for the natural history of the disease, the expanded use of ART, including various heterogeneities between individuals, in order to inform the public health community about the benefit of a prophylactic HIV vaccine.

## Methods

### Mathematical model

**Figure 1:**
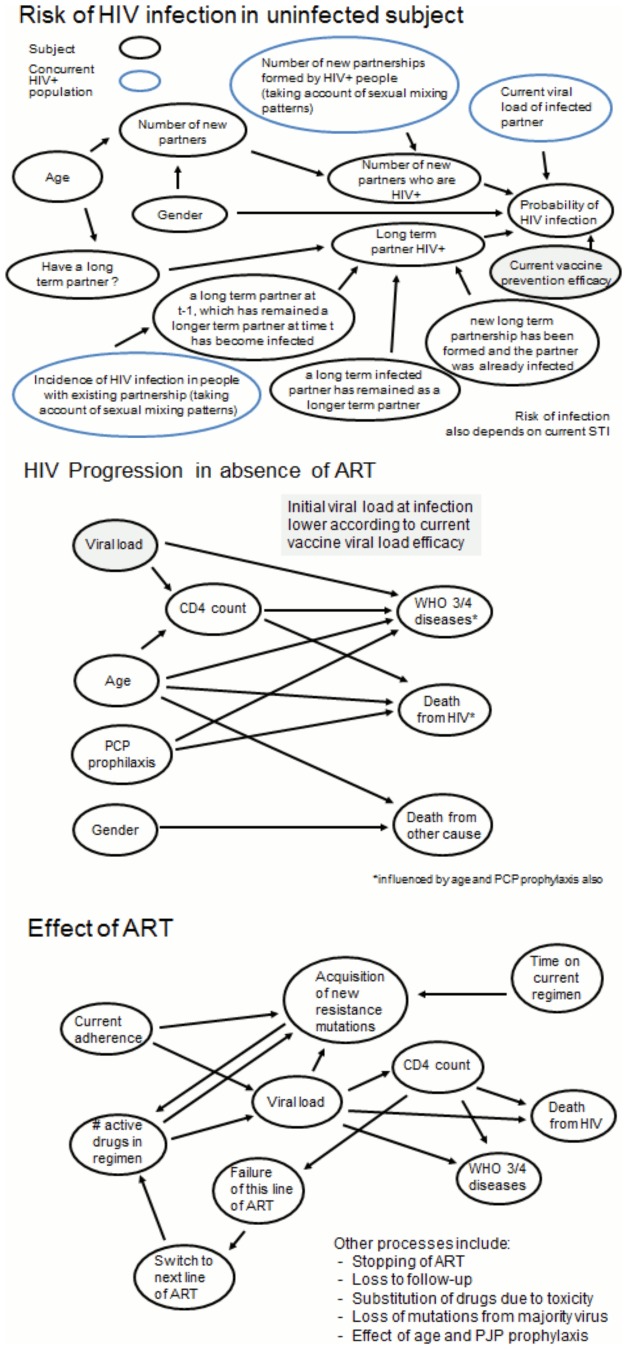
Summary of key variables and main influences in model. Effects of vaccine are illustrated in grey.

The HIV Synthesis Heterosexual Transmission Model is an individual-based stochastic model of heterosexual transmission, progression and treatment of HIV infection, described briefly in File S1, with full model details in previous supplementary materials [4], [5]. The basic model structure is shown in Figure 1. All variables in the model are updated in 3 month periods. The model includes an age-structure and the sexual risk behaviour is modelled as the number of condomless-sex short term partners and presence of a condomless-sex long-term partner in each period.

For each short or long-term partner that a subject has in a 3 month period, the probability that the partner is infected is calculated. For a short-term partner this depends on the age- and sex- specific prevalence of HIV amongst those in short-term partnerships. For a long-term partner, the HIV infection, diagnosis and ART status is tracked over time; in any 3 month period an uninfected long-term partner may become infected, dependent on the age-specific HIV-incidence in the population, a new long-term relationship with an infected short-term partner may form or a relationship with an infected long-term partner may continue. For exposure to each infected short- or long-term partner in the time period, the probability of transmission depends on the viral load level of the partner (for short-term partners this is obtained by sampling from the distribution of viral load levels in partnerships formed by HIV infected people, accounting for gender and age, and for long-term partners this depends on diagnosis and use of ART, estimated from the population of HIV-infected subjects), on the estimated risk of transmission at that viral load, gender, presence of a concurrent sexually transmitted infection in the subject and for male subjects on whether or not they are circumcised. If a subject is infected the probability of transmitted drug resistance depends on the viral load of the partner who infected the subject and viral load-specific population levels of resistance. For HIV-infected subjects variables modelled include: primary infection, CD4 count, viral load, presence of resistance, HIV diagnosis, care and treatment and adherence to treatment.

Using a single set of parameter values, we generated an epidemic from 1989–2025. Vaccine introduction scenarios are considered from 2025 (when it is believed a vaccine could be available, based on current status of the field and classical development timelines). We keep the same set of parameter values for the natural history of HIV, sexual risk behaviour and HIV management and treatment but make multiple runs for each vaccine implementation scenario and take means, to minimize stochastic effects on outcomes.

### Epidemic/programmatic scenario modelled

Our model was not specifically calibrated to data from any one country or region but broadly reflects historic levels of incidence and prevalence in settings in southern Africa [6]. ART was assumed introduced in 2003 with choice of drugs and monitoring strategy over the period to the present consistent with WHO guidelines of the time.

### Vaccine scenarios

Prevention efficacy of 0%, 30%, 50% and 90% were considered. Efficacy in reducing viral load set point at infection in those infected (with consequent effects on CD4 count decline and probability of viral suppression on ART as well as on infectivity) was 0.0, 1.0 or 2.0 log10 copies/mL. We focused on comparisons of outcomes of 8 different vaccine efficacy scenarios defined according to the efficacy of the vaccine in preventing infection and its efficacy in reducing viral load in infected vaccinees as follows: (i) prevention efficacy 0%, viral load efficacy 0.0 log_10_, (ii) prevention efficacy 30%, viral load efficacy 0.0 log_10_, (iii) prevention efficacy 50%, viral load efficacy 0.0 log_10_, (iv) prevention efficacy 90%, viral load efficacy 0.0 log_10_, (v) prevention efficacy 0%, viral load efficacy 1.0 log_10_, (vi) prevention efficacy 0%, viral load efficacy 2.0 log_10_, (vii) prevention efficacy 50%, viral load efficacy 1.0 log_10_, (viii) prevention efficacy 90%, viral load efficacy 2.0 log_10_. These 8 vaccine efficacy scenarios do not represent all possible permutations of vaccine prevention and viral load efficacy but were chosen *a priori* as a set of scenarios of particular interest.

Duration of vaccine effect was assumed to be 2 or 5 years, with the possibility of a tapered effect such that there is a linear decrease in efficacy down to half the initial efficacy by the end of the duration of the vaccine effect, i.e. efficacy was reduced by 50% by the end of the 2 or 5 year period. While we primarily considered that any vaccine effect would apply to all infected vaccinees, we also considered the possibility that each of the effects would only be present in half of those vaccinated. Considering uptake and coverage, we assumed a three dose schedule with the first 2 doses within the same 3 month period and the 3^rd^ dose 6 months later. Completion of the second dose, given the first was administered was assumed to be 98% (assumed no efficacy if only first dose administered), while completion of the dose after 6 months amongst those with the first two doses was assumed to be 94% (50% reduction in vaccination efficacy assumed if only first two doses administered). Current vaccination programmes for HPV vaccines will help to further inform these assumptions [7]. Vaccination at age 10 or age 15 were considered, with a rate of vaccination per 3 months of 0.2 or 0.3 amongst those age 10–12 or 15–17 (depending on the target age for vaccination), with a maximum coverage in this age group of either 40% or 70% (when rate of uptake is 0.2 only 93% of this maximum coverage achieved in 3 year vaccination period; 99% when rate is 0.3). We considered the possibility that there might be a 5 year catch-up program in adults aged 18–30 when the vaccine was first introduced, covering 50% of the population of that age. We also considered a scenario in which the vaccine was only provided to those at higher risk – defined as those having condom-less sex with a short-term in the past year. We considered that there may be a booster every 2 years or 5 years, depending on the duration of the assumed vaccine effect, up to age 30 or age 50. We assumed that only either 25%, 50% or 80% of people who had the first three vaccine doses would take these subsequent boosts (i.e. 75%, 50% or 20% of people vaccinated would not have any subsequent boost). While we generally assumed that those who took any booster would take all of them we also considered a scenario in which completion rates diminished over time such that only 80% of those who had a given boost then had the next boost. We conservatively assumed that vaccines that have an effect on viral load do not affect the infectivity of the infected vaccinee during primary infection, due to the fact that early high levels of viral replication are not greatly affected by the HIV-specific immune response [8].

We initially considered main results for our base scenario (vaccination at 15, with a rate of vaccination per 3 months of 0.3 amongst those age 15–17 (and a 5 year catch-up program amongst adults age 18–30), with a maximum coverage (in 15–17 year olds) of 70%, and with regular boosters every 5 years (the assumed duration of vaccine effect) to age 50, with 80% of people vaccinated then being adherent to all these subsequent boosts). In sensitivity analyses, we explored the effect of varying the vaccine or implementation characteristics in various ways, leaving all other parameters fixed.

### Multivariable uncertainty analysis

We also conducted a multivariable uncertainty analysis in which we simultaneously and independently sampled underlying model parameter values (for the natural history of HIV, sexual risk behaviour, HIV diagnosis, management and treatment) as specified in File S1, and generated the HIV epidemic from 1989–2025. For each of 500 model runs, from 2025–2060 we kept the sampled model parameter values fixed and generated the HIV epidemic for each of the 8 vaccine scenarios.

## Results

We first describe the characteristics of the modelled population in 2025, the year which we assumed a vaccine to be introduced (Table 1). The HIV prevalence is 25%, where 77% of people living with HIV are diagnosed, 71% of those diagnosed are under care, and 61% of the entire HIV infected population is on ART. This is not intended to correspond to one specific population in southern Africa but the profile is broadly in line with epidemic and programme data from the region.

**Table 1:** Characteristics of the population up to and at baseline (year 2025).*

HIV prevalence	1989	2%
	1990	7%
	1995	21%
	2000	25%
	2005	26%
	2010	28%
	2015	28%
	2020	27%
	2025	25%
Incidence of HIV (/100 person years)	1990	6.6
	1995	4.5
	2000	2.7
	2005	2.2
	2010	2.3
	2015	1.7
	2020	1.6
	2025	1.3
Death rate 2020–2025 (/100 person years)	Whole population	1.50
	HIV infected population	4.23
Percentage of people with HIV diagnosed		77%
Percentage of people with HIV diagnosed and under care		71%
Percentage on ART	Of whole population	15%
	Of HIV infected	61%
	Of HIV diagnosed	79%
Percentage with viral load <500 copies/mL	Of HIV infected	50%
	Of people on ART	79%
Percentage of all HIV infected with any drug resistance mutation		17%

*all values relate to 2025 unless stated.

**Figure 2:**
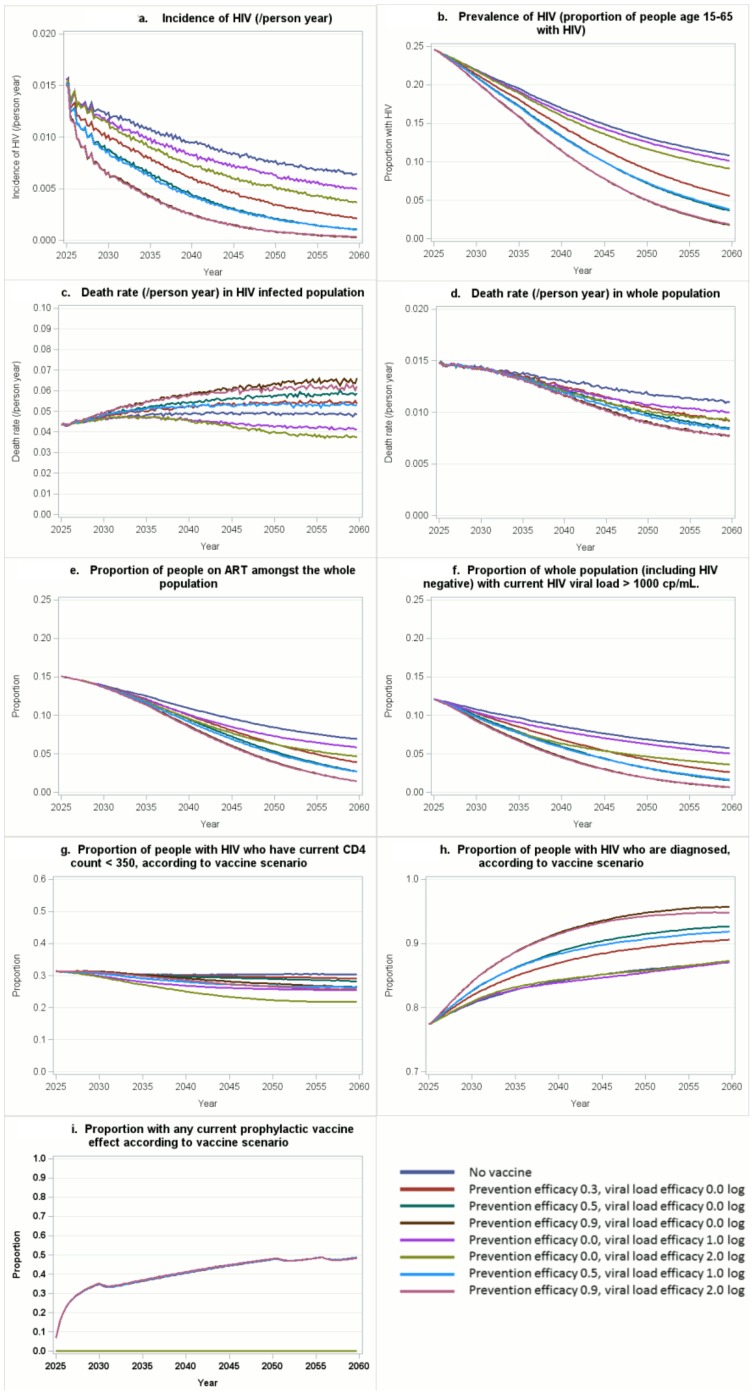
Predicted outcomes 2025–2060 of eight vaccine introduction scenarios in 2025. (i) prevention efficacy 0.0, viral load efficacy 0.0 log_10_, (ii) prevention efficacy 30%, viral load efficacy 0.0 log_10_, (iii) prevention efficacy 50%, viral load efficacy 0.0 log_10_, (iv) prevention efficacy 90%, viral load efficacy 0.0 log_10_, (v) prevention efficacy 0.0, viral load efficacy 1.0 log_10_, (vi) prevention efficacy 0.0, viral load efficacy 2.0 log_10_, (vii) prevention efficacy 50%, viral load efficacy 1.0 log_10_, (viii) prevention efficacy 90%, viral load efficacy 2.0 log_10_. All in the context of vaccination at 15, with a rate of vaccination per 3 months of 0.3 amongst those age 15–17 (and a 5 year catch-up program amongst adults age 18–30 covering 50% of the population of that age), with a maximum coverage (in 15–17 year olds) of 70%, and with regular boosters every 5 years (the assumed duration of vaccine effect) with 80% of people being adherent to these boosts.

We then considered projected outcomes from 2025–2060 according to vaccine efficacy characteristics. These are shown in Figure 2 and Figure S1 in File S1, in the context of the base scenario for vaccination. Differences in outcomes (HIV incidence, prevalence, percent of the whole population on ART, death rate in the whole population, and the percent of people with on-going vaccine effect) generally emerge gradually as vaccine coverage increases so in Table 2 we focus on differences from 2040 onwards by giving the mean of values of the outcomes over the period from 2040–2060. The proportion of the adult population vaccinated and with some on-going vaccine effect (i.e. vaccinated previously and still within the period of vaccine efficacy) rises to over 20% by 2028, within 2–3 years of the program initiation, and thereafter increases gradually to a ceiling level of around 45% over 25 years (by 2050; see Figure S1q, S1r in File S1).

**Table 2:** Mean over 2040–2060 of the following outcomes: HIV incidence (per 1000 person years), prevalence (%), % of whole population on ART (not only HIV infected), death rate (in whole population; per 100 person years), % of population age 15–65 with on-going vaccine effect (i.e. vaccinated and up to date with boosters), 2040–2060, for base implementation characteristics.

Vaccine efficacy	0%	30%	50%	90%	0%	0%	50%	90%
Prevention viral load (log_10_)	0.0	0.0	0.0	0.0	1.0	2.0	1.0	2.0
Incidence	7.7	3.7	2.3	1.0	6.6	5.2	2.3	1.0
	*7.5–7.9*	*3.5–3.9*	*2.2–2.4*	*0.8–1.2*	*6.2–6.8*	*5.0–5.4*	*2.1–2.5*	*0.8–1.2*
Prevalence	13.5	9.5	7.7	5.6	12.9	12.0	7.8	5.7
	*13.4–13.6*	*9.4–9.6*	*7.5–7.9*	*5.4–5.8*	*12.8–13.0*	*11.9–12.1*	*7.7–7.9*	*5.5–5.9*
% on ART+	8.6	6.6	5.6	4.4	7.6	6.6	5.4	4.3
	*8.5–8.7*	*6.5–6.7*	*5.5–5.7*	*4.3–4.5*	*7.5–7.7*	*6.5–6.7*	*5.3–5.5*	*4.2–4.4*
Death rate+	1.19	1.06	1.01	0.93	1.09	1.03	0.98	0.92
	*1.17–1.21*	*1.04–1.08*	*0.99–1.03*	*0.91–0.95*	*1.07–1.11*	*1.01–1.05*	*0.96–1.00*	*0.90–0.94*
% of people with on-going vaccine effect	0%	45%	45%	45%	45%	45%	45%	45%
	*0%–0%*	*44%–46%*	*44%–46%*	*44%–46%*	*44%–46%*	*44%–46%*	*44%–46%*	*44%–46%*

Base scenario: Coverage 70%, boosting to age 50, with 80% completion rates to boosters, no tapering in effect over time, and with an adult catch-up program in 18–30 years covering 50% of the population of that age, rate vaccination  = 0.3/3mths, vaccine effect on VL in 100% of people, duration of vaccine effect 5 years, age of introduction 15. 95% CI shown in italics.

+ of entire population, including HIV uninfected.

### Impact on incidence and prevalence

Next we considered the impact of the potential vaccine on incidence and prevalence of HIV infection in the population. While due to a combination of ART, increased circumcision and previous changes in condomless sex a decline in HIV incidence is predicted to occur (Figure 2a) even without any vaccine introduction (prevention efficacy and viral load efficacy both 0, see line in blue in Figure 2a), the predicted decline is markedly greater if a vaccine with prevention efficacy is introduced. Notably, even for a vaccine with 30% prevention efficacy (and no effect on viral load) there is a substantial effect, with a 52% reduction in mean HIV incidence 2040–2060 (3.7 per 1000 person years compared with 7.7 per 1000 person years with no vaccine) and a projected 67% lower HIV incidence in 2060 compared with no vaccine introduction. Vaccines with 50% and 90% efficacy lead to predicted mean incidence in 2040–2060 of 2.3 and 1.0 per 1000 person years, respectively. There is also an effect of a vaccine with viral load efficacy but no direct prevention efficacy on mean HIV incidence in 2040–2060 (6.6 and 5.2 per 1000 person years for a vaccine that reduces viral load set point by 1.0 log and 2 log, respectively). Differences in trends in HIV prevalence (Figure 2b) according to vaccine efficacy characteristics tend to follow the differences in HIV incidence.

### Impact on death rate, ART use and other key outputs

We then considered predicted differences in death rate according to the vaccine introduction scenarios. The death rate for people with HIV (Figure 2c) is highest in the scenario with highest vaccine prevention efficacy considered (90%), and lowest where there is no prevention efficacy and the highest viral load efficacy considered (2.0 log reduction). This is due to the selection effects on the HIV infected population; because there are less new HIV infections the population living with HIV is composed mainly of people who have been infected for a longer while and so are more likely to die. Figure 2d shows the death rate in the whole population, including infected and uninfected, which captures benefits of both prevention efficacy and viral load efficacy. Both forms of efficacy have a distinct beneficial effect on mortality in the population, although with a prevention efficacy of 90% there is little additional impact of a 2 log viral load efficacy.

Another issue to consider, are predicted changes in the use of ART. There are accompanying large reductions in the proportion of the whole adult population on ART (Figure 2e) which broadly follow the trends in HIV prevalence. Figure 2f shows the proportion of the whole adult population with viral load >1000 copies/mL, indicating the effects of the two types of vaccine efficacy on the number of infectious people in the population. HIV incidence is driven by this population of infectious people, and the curves follow a similar pattern to those for HIV incidence.

Predicted trends in the proportion of HIV positive people with CD4 count 350 are shown in Figure 2g. This proportion is highest where no vaccine is introduced and lowest where a vaccine with viral load efficacy, but no prophylactic efficacy, is introduced. The proportion of people with HIV who are diagnosed (Figure 2h) is predicted to be highest for a vaccine with high prophylactic efficacy. Both of these trends relate to the overall changing breakdown of the future HIV positive population resulting from selection effects due to fewer new infections (particularly with vaccines with prophylactic efficacy) and/or lower viral load in those becoming infected (for vaccines with viral load efficacy). The degree of ongoing prophylactic vaccine efficacy is shown in Figure 2i.

### Analysis of sensitivity to assumptions on vaccine characteristics

We then explored the extent to which our main findings described above differed when various changes in the vaccine-related parameters and assumptions were made. Table 3 (with full details in Table S1 in File S1) shows the effect of several variations in the vaccine-related parameters and assumptions, with one factor varied at a time. The average proportion of people aged 15–65 with on-going vaccine efficacy in the years 2040–2060 is at its highest at 45% (the coverage values of 40% and 70% relate to the proportion of people in the target age range (10–12 or 15–17) who are vaccinated - aside from the initial adult catch-up program, those who are not vaccinated by age 18 are assumed not vaccinated at all in their lifetime). The different variations in effect all led to various degrees of reduced on-going vaccine impact and this was generally correlated as expected with the effect on incidence and the death rate in the whole population. The average percent with on-going vaccine effect was lowest across all scenarios with only 25% booster completion rate. For some variations, the modification of the effect on incidence was only small, such as when the rate of vaccination was decreased from 0.3 to 0.2 per 3 months, or when the duration of vaccine efficacy was reduced from 5 to 2 years. The reason for the small impact of the latter variation is that it is assumed that booster doses are given after 2 rather than 5 years, so there is little detrimental effect on vaccine coverage (44% vs 45% in the base scenario). Vaccination at age 15, rather than age 10, is associated with greater vaccine effects, presumably due to the fact that people vaccinated at age 15 have immediate benefits (because they are already potentially sexually active) while, with vaccination at age 10, efficacy may be lost before sexual activity commences.

**Table 3:** Mean over 2040–2060 of on-going vaccine effect, HIV incidence (per 1000 person years and death rate (/100 person years) in the whole population, according to variations in vaccine implementation characteristics.

	Difference from base scenario		Vaccine efficacy							
		Prevention viral load (log_10_)	0%	30%	50%	90%	0%	0%	50%	90%
			0.0	0.0	0.0	0.0	1.0	2.0	1.0	2.0
% with on-going vaccine effect	None (base)		0%	45%	45%	45%	45%	45%	45%	45%
	Boosting to age 30		0%	26%	26%	26%	26%	26%	26%	26%
	50% completion rates to boosters		0%	34%	34%	34%	34%	34%	34%	34%
	25% completion rates to boosters		0%	23%	23%	23%	23%	23%	23%	23%
	Coverage 40%		0%	30%	30%	30%	30%	30%	30%	30%
	No adult catch-up program		0%	40%	40%	40%	40%	40%	40%	40%
	Tapering in effect		0%	45%	45%	45%	45%	45%	45%	45%
	Vaccination age 10		0%	42%	42%	42%	42%	42%	42%	42%
	Rate vaccination 0.2/3mths		0%	43%	43%	43%	43%	43%	43%	43%
	Duration vaccine effect 2 years		0%	44%	44%	44%	44%	44%	44%	44%
	Booster uptake decrease		0%	35%	35%	35%	35%	35%	35%	35%
	Vaccine effect on VL in 50% of people		0%	45%	45%	45%	23%	23%	45%	45%
	Prophylactic vaccine effect in 50% of people		0%	23%	23%	23%	45%	45%	45%	45%
	Targeted at people having condom-less sex in past year with new partner		0%	32%	32%	32%	32%	32%	32%	32%
Incidence (/1000 person years)	None (base)		7.7	3.7	2.3	1.0	6.6	5.2	2.3	1.0
	Boosting to age 30		7.7	4.4	3.1	1.7	6.6	5.6	2.9	1.6
	50% completion rates to boosters		7.7	4.3	3.0	1.5	6.7	5.6	2.8	1.5
	25% completion rates to boosters		7.7	4.9	3.7	2.1	7.1	6.1	3.6	2.1
	Coverage 40%		7.7	4.7	3.3	1.7	6.8	5.9	3.2	1.7
	No adult catch-up program		7.7	4.3	3.0	1.6	6.6	5.5	2.9	1.6
	Tapering in effect		7.7	6.6	5.9	4.6	6.5	5.2	5.0	3.5
	Vaccination age 10		7.7	4.1	2.7	1.3	6.4	5.5	2.7	1.3
	Rate vaccination 0.2/3mths		7.7	3.9	2.5	1.1	6.3	5.3	2.4	1.1
	Duration vaccine effect 2 years		7.7	3.9	2.4	1.1	6.4	5.3	2.4	1.1
	Booster uptake decrease		7.7	4.0	2.7	1.3	6.5	5.4	2.6	1.3
	Vaccine effect on VL in 50% of people		7.7	3.7	2.3	1.0	7.1	6.3	2.3	1.0
	Prophylactic vaccine effect in 50% of people		7.7	5.3	5.2	4.1	6.4	5.2	4.2	2.7
	Targeted at people having condom-less sex in past year with new partner		7.7	4.3	3.0	1.4	6.5	5.6	2.8	1.5
Death rate in whole population (/100 person years)	None (base)		1.19	1.06	1.01	0.93	1.09	1.03	0.98	0.92
	Boosting to age 30		1.19	1.08	1.02	0.95	1.11	1.05	1.00	0.94
	50% completion rates to boosters		1.19	1.08	1.03	0.95	1.11	1.06	1.00	0.95
	25% completion rates to boosters		1.19	1.09	1.05	0.98	1.13	1.08	1.03	0.97
	Coverage 40%		1.19	1.09	1.04	0.96	1.12	1.07	1.02	0.95
	No adult catch-up program		1.19	1.10	1.06	1.00	1.12	1.07	1.04	0.99
	Tapering in effect		1.19	1.16	1.14	1.11	1.09	1.03	1.06	1.00
	Vaccination age 10		1.19	1.08	1.03	0.96	1.10	1.05	1.01	0.95
	Rate vaccination 0.2/3mths		1.19	1.07	1.01	0.94	1.09	1.04	0.99	0.93
	Duration vaccine effect 2 years		1.19	1.07	1.01	0.94	1.09	1.04	0.99	0.93
	Booster uptake decrease		1.19	1.07	1.02	0.94	1.10	1.04	0.99	0.93
	Vaccine effect on VL in 50% of people		1.19	1.07	1.01	0.93	1.14	1.10	0.99	0.93
	Prophylactic vaccine effect in 50% of people		1.19	1.12	1.10	1.05	1.09	1.03	1.03	0.96
	Targeted at people having condom-less sex in past year with new partner		1.19	1.09	1.03	0.96	1.12	1.06	1.01	0.95

Base scenario: Coverage 70%, boosting to age 50, with 80% completion rates to boosters, no tapering in effect over time, and with an adult catch-up program in 18–30 years covering 50% of the population of that age, rate vaccination  = 0.3/3mths, vaccine effect on VL in 100% of people, duration of vaccine effect 5 years, age of introduction 15. For each row, one characteristic is made different from the base scenario. 95% CI shown in Table S1, along with further comparisons.

### Multivariable uncertainty analysis

For all the results shown above be made a set of assumptions and used certain parameter values for our underlying model (described in Figure 1). We therefore explored what variation there is in our main results if these assumptions are varied. In this mutivariable uncertainty analysis we varied all the parameters as described by the distributions in File S1. For each simulation run we independently sampled from each of the distributions as described. This is in contrast to the one way sensitivity analysis above that focussed only on varying the vaccine-related characteristics, and also only one assumption was varied at a time. In this analysis we focussed on our main comparison of incidence rates between the vaccines with various characteristics, and present these in the form of incidence rate ratios. Table 4 shows the distribution of incidence rate ratios, compared with the no vaccine scenario, for the 8 vaccine efficacy scenarios. Generally, the effects were of similar magnitude across the 500 runs but there was some appreciable variation. For example, the median incidence rate ratio over 2040–2060 for a vaccine of 30% prevention efficacy (0.48 in our base case analysis) was 0.59 with a 90% uncertainty range of 0.41–0.79. Overall, our choice of parameter distributions used in these analyses were such that the vaccine effects we saw in our base case scenario were slightly greater than the median effect in the uncertainty analysis.

**Table 4:** Multivariable uncertainty analysis based on 500 runs.

Prevention efficacy	Viral load efficacy (log_10_)	Incidence rate ratio (vs. no vaccine) 2040–60	
		Median (90% uncertainty range) over runs in which parameters vary	Base case
30%	0.0	0.59 (0.41–0.79)	0.48
50%	0.0	0.39 (0.21–0.60)	0.30
90%	0.0	0.17 (0.10–0.37)	0.13
0%	1.0	0.87 (0.69–1.09)	0.86
0%	2.0	0.77 (0.56–1.05)	0.68
50%	1.0	0.38 (0.23–0.58)	0.30
90%	2.0	0.16 (0.09–0.40)	0.13

Variation in effect of vaccine on HIV incidence under parameter variation, sampling from distributions of parameter values given in Supplementary Methods and Results in File S1.

## Discussion

These results from a detailed individual-based simulation model of the potential effects of a partially effective HIV vaccine on epidemics in southern Africa, in the context of assumed continued expansion of ART use, suggests that even a vaccine with 30% preventative efficacy could make a substantial difference in the rate with which HIV incidence declines. The impact on incidence in relative terms is projected to increase over time, with a projected 67% lower HIV incidence in 2060 compared with no vaccine introduction, which reflects the additional population impact due to effects of herd immunity. A vaccine with 50% prevention efficacy (but no effect on viral load) is predicted to reduce incidence in our scenario to around 0.11% per year in 2060, compared with 0.64% per year without a vaccine being introduced. A vaccine with no prevention efficacy but which reduces viral load by 1 log is predicted to result in some modest (14%) reduction in mean HIV incidence in 2040–2060 and to lead to an 8% decrease in death rate in the whole adult population (mean 2040–2060), while if viral load is reduced by 2 log then these reductions are 32% and 13%, respectively (Table 2). These results all relate to our base case scenario (illustrated by the results presented in Table 1) which we considered to be realistic. However, results on the effect of vaccines on HIV incidence were robust in multivariable uncertainty analyses in which we considered a wide range of epidemic/programmatic situations by simultaneously sampling multiple parameters from distributions reflecting uncertainty in their value. Our model can be used in the future to predict vaccine effects in any specified implementation scenario.

While prediction of the absolute future incidence of HIV in the absence of a vaccine is not the primary focus on this work, it is notable that our modelling predicts a continued decline in HIV incidence over the coming years in the absence of any vaccine introduction, consistent with other modelling estimates [1]. We assume continuation of ART roll-out had some effect on the resulting incidence over time (Figure S1g in File S1). While we do not assume further general population reductions in condom-less sex in this period, the effects of the earlier reductions may still be playing out in the period under study. Also, we do continue to assume a reduction in condom-less sex as a result of HIV diagnosis (average 17% reduction with new partners and 31% reduction with primary partners; for this reason we did not explicitly incorporate the fact that people would likely need to be tested HIV negative before being vaccinated, because the resulting higher levels of HIV testing and diagnosis would add an indirect benefit of vaccination which is difficult to separate from the intrinsic effect). These are likely to be the main reasons behind the continued predicted decline in incidence. If the ART roll out stalls then this would adversely affect future HIV incidence, as would any general population increases in condom-less sex. Such increases could occur as fear of HIV declines due to availability of ART or if focus and spending on prevention is diminished in the interests of expanded ART. Likewise there could be a specific tendency for people who are vaccinated to increase condom-less sex due to a feeling of protection from infection, which could make a major impact on net vaccine efficacy and could even potentially lead to an increase in incidence. Such “risk compensation” is a general concern for new prevention interventions and will be critical to study along with any vaccine trial and roll-out [9], [10]. We assume no substantial increase in male circumcision, with around 20% of men circumcised, or any use of antiretroviral-based pre-exposure prophylaxis, due to uncertainty over the extent of its future use. Another factor that could influence future incidence is the potential tendency for increased viral pathogenicity over time (which has been observed in some cohorts [11]), leading to continuation of the observed trend for lower initial CD4 count, causing earlier ART initiation and death in those not diagnosed, leading to greater reductions in incidence. Lastly, our model takes account of the development of drug resistance and its transmission, so the predicted improvements occur despite some increase in drug resistance [5], [12].

Various HIV transmission models, including some individual-based simulations models [13]–[15] have been used to analyse potential effects of HIV vaccines [13]–[32]. These have yielded insights consistent with some of our own observations. The finding that partially effective prophylactic vaccines, if effectively boosted so that vaccine efficacy levels are kept close to the maximal (albeit modest) effect, can have a substantial impact on reducing HIV incidence has been observed since early studies [18], [21], [23]. The potential effects of the ALVAC-HIV prime, AIDSVAX B/E antigen boost vaccine regimen in a trial in Thailand (with estimated 30% efficacy; RV144 trial [3] have been extensively modelled in various settings. While one round of vaccination was not predicted to have large effects, when on-going boosting at intervals was considered, predicted effects were substantial and the vaccine cost-effective [15], [29], [31], [32]. Some models have also taken account of the potential effect of a vaccine in reducing viral load, and hence infectivity and rate of CD4 count decline [17], [19], [20], [25], [33], but no individual-based stochastic models to our knowledge has previously taken account of all these factors in the context of detailed modelling of effects of ART. The one vaccine showed to have (partial) success in reducing HIV incidence, in the RV144 trial, did not appear to have any effect on viral load in infected vaccinees [3].

We have highlighted the strength of this modelling analysis but there are also limitations. By the nature of a modelling analysis, we made a number of assumptions, some of which are particularly uncertain considering that we project over almost a 50 year period. However, we did assess the variation in our key findings according to variations in these assumptions and found the basic results and conclusions to be stable. Our assumption of the level of vaccine uptake may seem relatively optimistic for a vaccine to be provided to adolescents but in context of high awareness of HIV and current levels of ART access we feel they are probably realistic considering that several countries in Africa have over 60% coverage of ART. Nonetheless, we also found a substantial effect if vaccine coverage was 40% rather than 70%.

In conclusion, introduction of a partially effective HIV vaccine would be predicted to make a substantial long term impact on HIV epidemics in southern Africa. Development of an HIV vaccine, even of relatively low efficacy, remains a critical public health goal.

## Supporting Information

File S1 Supplementary Methods and Results. (i) Brief description of HIV Synthesis Heterosexual Transmission Model for southern Africa, (ii) Epidemic scenario modelled, (iii) Vaccine and implementation characteristics, (iv) Full model details, (v) Parameters and distributions for uncertainty analysis, (vi) Table S1 (*Mean over 2040–2060 of the following outcomes: HIV incidence (per 1000 person years), prevalence (%), % of whole population on ART (not only HIV infected), death rate (in whole population; per 100 person years), % of uninfected population age 15–65 with an on-going vaccine effect (i.e. vaccinated and up to date with boosters), 2040–2060, according to vaccination efficacy and implementation characteristics. 95% CI shown in grey*), (vii) Figure S1 (*Predicted outcomes 2025–2060 of eight vaccine introduction scenarios in 2025: (i) prevention efficacy 0.0, viral load efficacy 0.0 log_10_, (ii) prevention efficacy 0.3, viral load efficacy 0.0 log_10_, (iii) prevention efficacy 0.5, viral load efficacy 0.0 log_10_, (iv) prevention efficacy 0.9, viral load efficacy 0.0 log_10_, (v) prevention efficacy 0.0, viral load efficacy 1.0 log_10_, (vi) prevention efficacy 0.0, viral load efficacy 2.0 log_10_, (vii) prevention efficacy 0.5, viral load efficacy 1.0 log_10_, (viii) prevention efficacy 0.9, viral load efficacy 2.0 log_10_. All in the context of vaccination at 15, with a rate of vaccination per 3 months of 0.3 amongst those age 15–17 (and a 5 year catch-up program amongst adults age 18–30 covering 50% of the population of that age), with a maximum coverage (in 15–17 year olds) of 0.7, and with regular boosters every 5 years (the assumed duration of vaccine effect) with 80% of people being adherent to these boosts. See footnote for full description of variable definition*).
(DOC)
