# Supplementary material for: Potential Future Impact of a Partially Effective HIV Vaccine in a Southern African Setting
Source: PLoS One. 2014 Sep 10;9(9):e107214. doi: 10.1371/journal.pone.0107214 (PMC4160197; doi:10.1371/journal.pone.0107214)
Supplement: File S1 — Supplementary Methods and Results. (i) Brief description of HIV Synthesis Heterosexual Transmission Model for southern Africa, (ii) Epidemic scenario modelled, (iii) Vaccine and implementation characteristics, (iv) Full model details, (v) Parameters and distributions for uncertainty analysis, (vi) Table S1 (Mean over 2040–2060 of the following outcomes: HIV incidence (per 1000 person years), prevalence (%), % of whole population on ART (not only HIV infected), death rate (in whole population; per 100 person years), % of uninfected population age 15–65 with an on-going vaccine effect (i.e. vaccinated and up to date with boosters), 2040–2060, according to vaccination efficacy and implementation characteristics. 95% CI shown in grey), (vii) Figure S1 (Predicted outcomes 2025–2060 of eight vaccine introduction scenarios in 2025: (i) prevention efficacy 0.0, viral load efficacy 0.0 log10, (ii) prevention efficacy 0.3, viral load efficacy 0.0 log10, (iii) prevention efficacy 0.5, viral load efficacy 0.0 log10, (iv) prevention efficacy 0.9, viral load efficacy 0.0 log10, (v) prevention efficacy 0.0, viral load efficacy 1.0 log10, (vi) prevention efficacy 0.0, viral load efficacy 2.0 log10, (vii) prevention efficacy 0.5, viral load efficacy 1.0 log10, (viii) prevention efficacy 0.9, viral load efficacy 2.0 log10. All in the context of vaccination at 15, with a rate of vaccination per 3 months of 0.3 amongst those age 15–17 (and a 5 year catch-up program amongst adults age 18–30 covering 50% of the population of that age), with a maximum coverage (in 15–17 year olds) of 0.7, and with regular boosters every 5 years (the assumed duration of vaccine effect) with 80% of people being adherent to these boosts. See footnote for full description of variable definition). (DOC) [file pone.0107214.s001.doc]

**File S1**

**Potential future impact of a partially effective HIV vaccine in a southern African setting in the context of continued ART roll out.**

**Supplementary Methods and Results**

**Contents Page**

Brief description of HIV Synthesis Heterosexual Transmission Model for southern Africa 2

Epidemic scenario modelled 2

Vaccine and implementation characteristics 2

Full model details 4

Parameters and distributions for uncertainty analysis 42

Table S1 47

Figure S1 52

***HIV Synthesis Heterosexual Transmission Model for southern Africa***

The HIV Synthesis Heterosexual Transmission Model is an individual-based stochastic model of heterosexual transmission, progression and treatment of HIV infection. All variables in the model are updated in 3 month periods. The model includes an age-structure and the sexual risk behaviour is modelled as the number of condomless-sex short term partners and presence of a condomless-sex long-term partner in each period. The HIV infection, diagnosis and ART status of long term condomless sex partners is tracked over time. In any given period, the probability of an uninfected person having a condomless-sex partner who is infected with HIV depends on their number of partners and on the prevalence of HIV amongst partnerships formed by those of the opposite gender, accounting for patterns of age mixing. Given exposure to an infected partner, the probability of transmission depends on the viral load level of the partner (obtained by sampling from the distribution of viral load levels in partnerships formed by HIV infected people, accounting for gender and age), on the estimated risk of transmission at that viral load, presence of a concurrent sexually transmitted infection and on gender. The model considers adherence to ART, effects of specific antiretroviral drugs on resistance acquisition and toxicity, as well as transmission of drug resistance. Full model details have been published (see refs 4, 5 in main manuscript).

***Epidemic scenario modelled***

We generated an epidemic starting in 1989 with a prevalence curve up to 2010 broadly consistent with those seen in southern African countries. The number of people simulated in each run is 100,000 – each one of these persons is at age 15-65 in some period between 1980 (the start of the simulation, even though HIV is not introduced until 1989) and 2060, but the population size in this age band at any one point is lower than 100,000 because, for example, people who will be 15 years old in 2050 are not included in the adult population until this date. ART was assumed introduced in 2003 with choice of drugs and monitoring strategy consistent with WHO guidelines of the time, so with tenofovir-based first line after 2010 and with viral load monitoring. For the main results, we use a single simulation run to 2025 based on a set of parameter values which we consider most plausible and thus generate a single epidemic scenario. After 2025, when vaccine introduction scenarios are considered, we keep the same set of parameter values but make multiple runs for each vaccine implementation scenario and take means, to minimize stochastic effects on outcomes.

***Vaccine and implementation characteristics***

We considered various characteristics of a vaccine and of its adherence and coverage. Prophylactic efficacy of 0%, 30%, 50% and 90% were considered. Efficacy in reducing viral load at infection in those infected (with consequent effects on CD4 count decline and probability of viral suppression on ART as well as on infectivity) was 0.0, 1.0 or 2.0 log10 copies/mL. Duration of vaccine effect was assumed to be 2 years or 5 years. While we primarily considered that any vaccine effect would apply to all infected vaccinees, we also considered the possibility that the effect would only be present in one half of those vaccinated. Considering uptake and coverage, we assumed a three dose schedule with the first 2 doses within the same 3 month period and the 3^rd^ dose 6 months later. Completion rates of the second dose, given the first was administered was assumed to be 98% (assumed no efficacy if only first dose administered), while completion rates of the dose after 6 months amongst those with the first two doses was assumed to be 94% (50% reduction in vaccine efficacy if only first two doses administered). Vaccination at age 10 or age 15 were considered, with a rate of vaccination per 3 months of 0.2 or 0.3 amongst those age 10-12 or 15-17 (depending on the target age for vaccination), with a maximum coverage in this age group of either 40% or 70% (when rate of uptake is 0.2 only 93% of this maximum coverage achieved in 3 year vaccination period; 99% when rate is 0.3). We considered the possibility that there might be a 5 year catch-up program in adults aged 18-30 when the vaccine was first introduced, covering 50% of the population of that age. We also considered a scenario in which the vaccine was only provided to those at higher risk – defined as those having condom-less sex with a new partner in the past year. We considered that there may be a booster every 2 years or 5 years, depending, on the duration of vaccine effect, up to age 30 or age 50. We assumed that only either 25%, 50% or 80% of people who had the 3 first vaccine doses would take these subsequent boosts (i.e. 75%, 50% or 20% of people vaccinated would not have any subsequent boost). While we generally assumed that those who took any booster would take all of them, we also considered a scenario in which completion rates diminished over time such that only 80% of those who had a given boost then had the next boost, etc. We generally considered that vaccine efficacy would be the same for the whole 2 or 5 year period that it covered, but we also explored the effect of a linear tapering in vaccine efficacy such that the efficacy was reduced by 50% by the end of the 2 or 5 year period. We conservatively assume that vaccines that have an effect on viral load do not affect the infectivity of the infected vaccinee during primary infection, due to the fact that early high levels of viral replication are not greatly affected by the HIV-specific immune response.

We focus on comparison of outcomes of 8 different vaccine introduction scenarios defined according to the efficacy of the vaccine in preventing infection and its efficacy in reducing viral load in infected vaccinees as follows: (i) prevention efficacy 0%, viral load efficacy 0.0 log_10_, (ii) prevention efficacy 30%, viral load efficacy 0.0 log_10_, (iii) prevention efficacy 50%, viral load efficacy 0.0 log_10_, (iv) prevention efficacy 90%, viral load efficacy 0.0 log_10_, (v) prevention efficacy 0%, viral load efficacy 1.0 log_10_, (vi) prevention efficacy 0%, viral load efficacy 2.0 log_10_, (vii) prevention efficacy 50%, viral load efficacy 1.0 log_10_, (viii) prevention efficacy 90%, viral load efficacy 2.0 log_10_.

We initially consider main results for our base scenario (vaccination at 15, with a rate of vaccination per 3 months of 0.3 amongst those age 15-17 (and a 5 year catch-up program amongst adults age 18-30 covering 50% of the population of that age), with a maximum coverage (in 15-17 year olds) of 70%, and with regular boosters every 5 years (the assumed duration of vaccine effect) to age 50, with 80% of people vaccinated then being adherent to all these subsequent boosts). We then conducted one way sensitivity analyses to explore the effect of varying the vaccine or implementation characteristics. We also conducted a multivariable uncertainty analysis in which we simultaneously and independently sampled parameter values from distributions (as specified in Supplementary Methods), which involved generating the entire HIV epidemic from 1989-2060 in each of 500 model runs. For each such run we compared outcomes over the 8 vaccine scenarios.

**HIV Synthesis Heterosexual Transmission Model**

**Full Model Details**

Note that in this material we mention distributions used for parameter values. For our main analysis we use fixed values and the distributions are used for multivariable uncertainty analysis only. The parameter distributions are shown below.

1. Demographic model

The intention is to simulate a range of epidemics similar to those seen in southern Africa with variations in sexual risk behaviour patterns (including in the extent of sex between males and female sex workers), dates of the start of the epidemic and of population level change in sexual behaviour.

**1. 1. General population death rates and determination of age at 1985**

The model runs for 80 years from 1980, with variables updated in 3 month periods. Each run of the simulation program creates 100,000 simulated people.

Age specific death rates for uninfected people (based on death rates in South Africa in 1997 – before the significant impact of HIV-related deaths) are as follows:-

Age group Annual death rate

--------------------------------------------

Males

15-19 0.00200

20-24 0.00320

25-29 0.00580

30-34 0.00750

35-39 0.00800

40-44 0.01000

45-49 0.01200

50-54 0.01900

55-59 0.02500

60-64 0.03500

65-69 0.04500

70-74 0.05500

75-79 0.06500

80-84 0.10000

>85 0.40000

Females

15-19 0.00150

20-24 0.00280

25-29 0.00400

30-34 0.00400

35-39 0.00420

40-44 0.00550

45-49 0.00750

50-54 0.01100

55-59 0.01500

60-64 0.02100

65-69 0.03000

70-74 0.03800

75-79 0.05000

80-84 0.07000

>85 0.15000

--------------------------------------------

These death rates are modified by a factor 1.5 for smokers and by 0.75 for non-smokers. This is due to the known effects of smoking on all cause mortality (1).

The initial age distribution for both males and females is determined on the basis of the following distribution.

Probability

Age group of being in age

group in 1980*

-------------------------------------------

-65-14 0.72572

15-24 0.06592

25-34 0.06041

35-44 0.05490

45-54 0.04939

55-64 0.04388

--------------------------------------------

* the actual age of a person in a given group in 1980 is determined by sampling from a Uniform distribution.

This distribution is chosen such that in the absence of HIV, given the death rates above, the population size increases over time (and hence the proportion of younger age also increases). Thus around 72% of simulated people have an age below 15 in 1980. The only variable that is modelled and updated up to reaching the age of 15 (when becoming potentially sexually active) is age itself.5 The “youngest” person in 1980 is age -65 (i.e. will be born in 2045 and reach age 15 in 2060, when the modelled period ends.

2. Model of sexual behaviour and risk of HIV acquisition

Sexual behaviour is characterized by two variables representing, respectively, the number of short term condomless sex partners and whether the person has a current long term condomless *sex* partners in the 3 month period. The status of long term partners is tracked over time (i.e. if they are infected, diagnosed, on ART, etc). Short term partners are not tracked over time, in that if a person has a short term partner in time period t who is infected with HIV, this is independent of the probability that any short term partner in time t+1 is infected with HIV.

**2.1. Determination of number of short term partners at period t**

Numbers of short term partners in a given period was generated at random, according to which of four sexual behaviour groups the person was in for this period. Changes in the sexual behaviour group from t-1 to t were determined by transition probabilities between 4 groups: (i) no short term unprotected partners in 3 month period, (ii) 1 short term partner, (iii) medium number of short term partners, and (iv) high number of short term partners. Transition probabilities
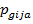
 of moving from partner group i at t-1 to partner group j at t are given by


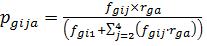


where g = 0,1 for males, females, respectively, and a = 1-10 for age groups 15-, 20-, 25-, 30-, 35-, 40-, 45-, 50-, 55-, 60-, respectively. Values of
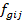
and
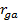
 are given in Tables 1 and 2, respectively, and if j=1 then
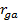
=1.

Values of
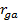
are modified at time t by a factor 0.2 if the subject has a current AIDS defining disease and by a factor *ch_risk_diag_newp* (distribution: Beta(10,4)) if the subject is diagnosed with HIV (sqrt(*ch_risk_diag_newp* from 6 months after diagnosis). In addition, there is a person-fixed modification factor (*p_rred_p* distribution: uniform(0.1,0.4)). For a proportion *p_rred_p* of men and 1.5.*p_rred_p* of women, values of
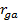
are modified by a factor 0.1, to reflect the fact that a proportion of people experience only very low sexual risk activity in their life.

Actual transitions between groups were determined by random sampling. For the first two groups the number of partners in the period is given (i.e. no short term partners, 1 short term partner, respectively). When a person was in the medium short term partners group the number of partners was determined by sampling from a Poisson(*highsa*), where *highsa* (distribution: uniform(1.5,5.5)) is a parameter sampled for each epidemic run. When in the high short term partners group the number of partners was determined by sampling from a Poisson(2) distribution and multiplied by the parameter *swn (*distribution: gamma(10,20), where *swn* is a parameter sampled for each epidemic run. Details of distributions of parameter values are given at the end of this document.

**2.2. Determination of having a long term (unprotected sex) partner at period t**

Note that only unprotected sex partnerships are modelled. Thus if a person has a long term partner but condoms are used on all occasions of sexual intercourse then this is not counted as having a long term partner.

At each period people with no current long term partner have age-dependent probabilities of having a new long term partner is dependent on parameter *eprate* and given by: age 15-24, p= *eprate*; age 25-34, p= *eprate*; age 35-44, p= *eprate/2*; age 45-54, p= *eprate/3*; age 55-64, p= *eprate/5*. (*eprate* distrbution: lognormal(ln0.1,0.25^2^))

At the time a long term partnership is started, it is classified into 3 duration groups, each with a different tendency to endure. The percent of people in each group is dependent on age and is shown in Table 3.

At time period, t, for people with a long term partner, the probability of the condomless sex partnership continuing is (1-(0.25 / *ch_risk_beh_ep*)) if duration category is 1, is (1-(0.05 / *ch_risk_beh_ep*)) if duration category is 2, and (1-(0.02 / *ch_risk_beh_ep*)) if duration category is 3, where *ch_risk_beh_ep* is a parameter conveying the population level change in sexual behaviour with long term partners that can occur at a certain date (triggered by an HIV prevalance above a certain threshold) (distribution *ch_risk_beh_ep*: Beta(7,2)) Further, this probability is reduced by a factor *ch_risk_diag* in the 3 month period after a partner’s diagnosis, if a partner has HIV and is diagnosed. (Distribution *ch_risk_diag* Beta (10,5)).

Note also that levels of sexual behaviour, in terms of numbers of short term partners and the probability of a long term partner are essentially determined by the levels of such sexual behaviour required in order to produce an epidemic as described, given rates of transmission with unprotected sex partners. Sexual behaviour tends to be under-reported particularly in women and higher levels of behaviour have to be assumed both to be consistent with levels of risk behaviour reported in men, and to generate an epidemic of the proportions observed (e.g. 2, 3). Nonetheless, reported sexual behaviour, particularly in terms of differences by age in males and females have been referred to (4, 5).

**Population level change in sexual behaviour**

There is assumed to be a general average reduction in condomless sex after HIV prevalance reaches a certain high value which is a threshold sampled from a distribution for each epidemic (*prev_threshold_rb_change*  distribution: Uniform(0.1,0.4))

**2.3. Determination of number of short term partners who are HIV infected at time t**

For each short term partner that a subject has at time t, the probability that the partner is infected is calculated. This is dependent on the prevalence of HIV in those of the opposite gender, taking consideration of age mixing. If the subject is of gender g and age group a, then for each short term partner the first step is to determine by sampling at random, the age group of the short term partner,
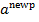
(in fact, for simplicity, all short term partners at time t are assumed to be in this same age group). The gender and age mixing probabilities used are given by values in Table 4.

Then, for the given partner (of gender 1-g and age group *a^newp^*), the risk that the partner is infected is then given by


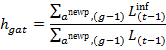


where
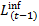
 is the total number of infected short term partners at time (t-1), and
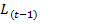
 is the total number of short term partners at time t-1. The numerator is therefore the total number of infected short term partnerships of the opposite gender in age group *a^newp^.*

Since we assume that all short term partners at time t are in this same age group, the total number of infected short term partners that the subject has at time t,
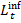
, is then given by


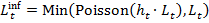


The distribution of numbers of partners by age and gender, before introduction of HIV, is illustrated for one example epidemic in Table 5.

**2.4. Determination of probability that a long term partner is HIV infected at time t**


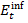
indicates whether the subject has a long term (unprotected sex) partner who is infected (
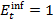
 if infected, else
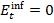
). A long term partner at time t can be infected either because (i) a new long term partnership has been formed and the partner was already infected, (ii) because a long term partner at t-1, which has remained a long term partner at time t, has become infected, or (iii) because a long term infected longer partner has remained as a long term partner.

For (i):


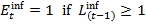
 (i.e. if the subject had a short term partner at time t-1 who was infected then it is assumed that the new long term partner is infected)

For (ii):

The probability that a long term partner of a subject of age group a and gender g becomes infected is derived from the HIV incidence at t-1 for age group a (i.e. the same age group) and gender 1-g,
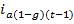
 among the sexually active population, either with a CLLT partner or at least one CLST partner (which is given by the number of subjects newly infected in age group at time *t-1* divided by the number of HIV-uninfected subjects in age group at *t-1*, who had condom-less relationships, either CLLT or CLST)


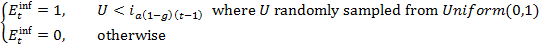


In order to maintain balance, for each gender, between the number of uninfected people with a long term partner who is infected, and the number of infected people with a long term partner who is uninfected, this incidence
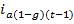
 is modified at time t dependent on the degree of balance at time t-1.

For (iii):

If
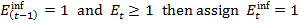


**2.5. Determination of the risk of infection from a short term partner**

For each HIV infected short term partner of a subject of gender g and age group a the viral load group, v, of the partner is obtained by sampling from the viral load distribution of those of the opposite gender. Thus we sample from Uniform(0,1), where the probability of the partner having viral load in group v is given by


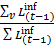


where the numerator is the total number of short-term partnerships had by infected people in viral load group v and the denominator is the total number of short-term partnerships had by infected people (in any viral load group).

Viral load groups are:

(1) < 2.7 log cps/mL

(2) 2.7-3.7 log cps/mL

(3) 3.7-4.7 log cps/mL

(4) 4.7-5.7 log cps/mL

(5) > 5.7 log cps/mL

(6) primary infection.

Once the viral load group, v, of the infected partner is determined, the probability, t_v_, of the subject being infected by the partner is then given according to: t_1_ = Normal (*tr_rate_undetec_vl*,0.000025^2^), t_2_ = Normal (0.01,0.0025^2^), t_3_ = Normal (0.03,0.0075^2^), t_4_ = Normal (0.06,0.015^2^), t_5_ = Normal (0.1,0.025^2^), t_6_ = Normal (*tr_rate_primary*,0.075^2^). These are based on ref 6 and are the rates for a longer term partner. The transmission rate for a short term partner is multipled by fold_tr_newp (distribution: beta(6,10)) due to the assumed lower number of sex acts. These probabilities are increased by *fold_change_w*-fold (distribution: lognormal( ln1.5,0.3^2^)) for female subjects aged > 20, by 2-fold for female subjects aged < 20, and by *fold_change_sti*-fold (distribution: lognormal( ln3,0.3^2^)) if the person has an existing STI (risk of a new STI in any one three month period is given by the number of short term unprotected partners / 20 (or 1 if > 20 short term partners)) (7-9).

Uncertainty in the transmission rate in groups (1)-(5) above is incorporated by sampling for each epidemic (run of the model program) from a distribution (lognormal( ln1.0,0.3^2^)) for a parameter *fold_tr* , by which the transmission rate is multiplied.

Uncertainty in the rate of transmission in primary HIV infection is incorporated by sampling a value of the parameter *tr_rate_primary* for each epidemic (distribution: beta(25,80)), and uncertainty in the transmission rate when plasma viral load is < 500 cps/mL is included by sampling the value for parameter *tr_rate_undetec_vl* (distribution: min(1, lognormal( ln0.0001,3^2^)))

We assume (for 90% of runs) that super-infection can occur(i.e. a person can be reinfected with HIV with consequent risk of acquiring new mutations).

Realization of whether the subject is infected by each short term partner is determined by sampling from Uniform(0,1).

**2.6. Determination of the risk of infection from a long term partner**

Infected long term partners at time t are classified by whether they are in primary infection (if infection occurred at t-1), whether they are diagnosed with HIV, whether they are on ART, and whether their current viral load is < 2.7 cps/mL or not. The proportion of long term partners with HIV who have HIV diagnosed at time t,
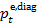
, is determined with reference to the difference,
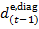
, in the proportion of subjects with HIV who are diagnosed,
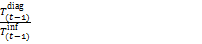
 and
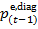
;

i.e.
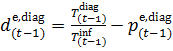


where
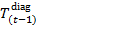
 is the total number of subjects diagnosed with HIV at time t-1and
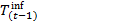
 is the total number of subjects with HIV (diagnosed and undiagnosed) at time t-1.


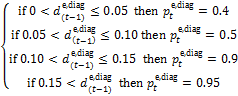


The proportion of those diagnosed who are on ART, and the proportion of those on ART who have viral load < 2.7 log cps/mL are determined in a similar manner. In this way the proportions diagnosed with HIV, on ART, and with current viral load is < 2.7 cps/mL are kept similar for the long term partners as in the simulated subjects themselves.

Risk of infection from a long term infected partner is determined by Normal (*tr_rate_primary*, 0.075^2^) if the existing partner is in primary infection (ie. infected at t-1), Normal (*tr_rate_undetec_vl*, 0.000025^2^) if the existing partner has viral load < 2.7 cps/mL, and Normal (0.05, 0.0125^2^) otherwise.

**2.7. Transmitted resistance**

The viral load group of the person who infected the subject is known, as indicated above. For a subject infected by a person in viral load group v the probability of a resistance mutation being present in the infected person is given by


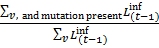


where
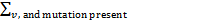
is the sum over all partnerships had by HIV-infected people in viral load group v for whom a resistance mutation is present in majority virus and
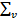
is the sum over all HIV-infected subjects in viral load group v. Again, realization of whether the subject is infected by a person with at least one resistance mutation in majority virus is determined by sampling from Uniform(0,1).

For subjects infected from a source partner with a resistance mutation, the probability that a specific mutation, m, is present in the source is given by


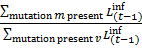


Where
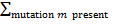
is the sum over all HIV-infected subjects with mutation m present in majority virus and
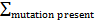
is the sum over all HIV-infected subjects with at least one resistance mutation in majority virus.

If a given resistance mutation, m, is present in the source partner, the probability that the mutation is both transmitted and survives in the subject (i.e. that its presence will affect future response to drugs for which the mutation confers reduced sensitivity) is mutation specific, as shown in Table 6.

We consider uncertainty in the extent to which transmitted resistance mutations are effectively immediately lost (even from minority virus) by sampling from a distribution for parameter *res_trans_factor* (distribution:

lognormal(1.0, 0.3^2^)).

**Loss from majority virus of transmitted mutations**

There is a probability per 3 months of loss of persistence of transmitetd mutations from majority virus to minority virus (same for each mutation) *rate_loss_persistence* (distribution: lognormal( ln004,0.3^2^)).

**2.8. Example results from epidemics simulated used modal values of parameter distributions**

To illustrate some features of the epidemics generated, Table 7 shows the proportion of people with at least one (at least two) condomless sex partner in the past year by HIV status and year using modal values for parameters. Table 8 shows the proportion of new infections that have been acquired from a person in primary HIV infection by year, and the proportion of new infections that have been acquired from a long term partner by year. Table 9 shows HIV prevalence by age and gender and calendar year.

**Table 1.** Values of
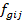
(values determining probability of transitioning between short term partner risk behaviour groups)

--------------------------------------------------------------------------------------------------------------------

Short term partners Short term partners group in period t

group in period t-1

0 1 medium high

Poisson Poisson

mean *highsa** mean 2

x *swn**

--------------------------------------------------------------------------------------------------------------------

**Males**

0 0.89 0.08 0.03 0.00

1 0.80 0.15 0.05 0.00

medium 0.35 0.27 0.38 0.00

high --- --- --- ---

**Females**

0 0.93 0.05 0.02 0.00025

1 0.86 0.11 0.03 0.0005

medium 0.54 0.08 0.38 0.001

high 0.05 0.05 0.10 0.800

-----------------------------------------------------------------------------------------------------------------------

* *highsa* distribution uniform(1.5,5.5), *swn* distribution gamma (10,20)

**Table 2.** Values of
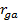
(factor determining relative level of sexual risk activity)

------------------------------------------------------------------------------------------

Age group Males females

(a=1,10) (g=1) (g=2)

------------------------------------------------------------------------------------------

15- 0.60 1.60

20- 0.60 1.60

25- 1.00 1.00

30- 0.80 0.80

35- 0.65 0.50

40- 0.50 0.35

45- 0.40 0.10

50- 0.35 0.05

55- 0.25 0.04

60- 0.15 0.02

------------------------------------------------------------------------------------------

**Table 3.** Percent of newly formed long term partnerships classified into each of three duration groups, each of which has a different tendency to endure (higher class, more durable).

Age 1 2 3

-----------------------------------------------------------------------------------------------

15-44 30% 30% 40%

45-54 30% 50% 20%

55-64 30% 70% 0%

-----------------------------------------------------------------------------------------------

**Table 4**. Sexual mixing by age and gender. The proportion of short term partnerships formed by men in age group a_m_ which are with females of age group a_f_ and the proportion of short term partnerships formed by females in age group a_f_ which are with men of age group a_m_.

Females

Age group (a_f_)

Males

Age group 15-24 25-34 35-44 45-54 55-64

(a_m_)

----------------------------------------------------------------------------------------

15-24 0.865 0.11 0.025 0.00 0.00

25-34 0.47 0.43 0.10 0.00 0.00

35-44 0.30 0.50 0.20 0.00 0.00

45-54 0.43 0.30 0.23 0.03 0.01

55-64 0.18 0.18 0.27 0.27 0.10

----------------------------------------------------------------------------------------

Males

Age group (a_m_)

Females

Age group 15-24 25-34 35-44 45-54 55-64

(a_f_)

----------------------------------------------------------------------------------------

15-24 0.43 0.34 0.12 0.10 0.01

25-34 0.09 0.49 0.30 0.10 0.02

35-44 0.03 0.25 0.34 0.25 0.13

45-54 0.00 0.00 0.05 0.25 0.70

55-64 0.00 0.00 0.00 0.10 0.90

----------------------------------------------------------------------------------------

**Table 5.** Sexual risk behaviour before introduction of HIV for one example epidemic using modal values for parameters (mean over 30 runs).

% with > 1 (>2) condomless sex partners (short or long term) in past year

Age group Males Females

-------------------------------------------------------------------------------------------------------------------------------------------------

15- 71% (29%) 75% (41%)

25- 85% (46%) 82% (36%)

35- 74% (31%) 67% (17%)

45- 65% (21%) 54% (4%)

55- 53% (10%) 45% (2% )

-------------------------------------------------------------------------------------------------------------------------------------------------

% with > 5, 10 short term condomless sex partners in past 3 months

Age group Males Females

-------------------------------------------------------------------------------------------------------------------------------------------------

15- 1.2% (0.02%) 2.6% (2.5%)

25- 2.0% (0.02%) 1.8% (1.8%)

35- 1.2% (0.01%) 0.8% (0.8%)

45- 0.8% (0.01%) 0.1% (0.1%)

55- 0.4% (0.00%) 0.1% (0.1% )

-------------------------------------------------------------------------------------------------------------------------------------------------

% with long term condomless sex partner

Age group Males Females

-------------------------------------------------------------------------------------------------------------------------------------------------

15- 48% 48%

25- 57% 57%

35- 48% 47%

45- 41% 41%

55- 35% 34%

-------------------------------------------------------------------------------------------------------------------------------------------------

**Alternative sexual behaviour model.**

In addition, an alternative sexual behaviour structure has also been developed, in which no woman or man has more than 10 short term partners in a 3 month period.

**Table 6.** Table of probabilities that for a given mutation present in the source partner the mutation is both transmitted and survives in the subject. (based on evidence from studies comparing distribution of resistance mutations between treated and antiretroviral naïve populations; e.g. 10, 11.

-------------------------------------------------------------------------

M184V 0.4

K65R 0.4

L74V 0.85

Q151M 0.85

Thymidine analogue mutations (TAMS) 0.85

NNRTI mutation 0.85

PI (lopinavir) mutations 0.85

(46, 82, 84, 90)

--------------------------------------------------------------------------

**Table 7.** Proportion of people with at least one (at least two) condomless sex partner in the past year by HIV status and year using modal values for parameters (mean over 30 runs).

1985 1990 1995 2000 2005 2010

------------------------------------------------------------------------------------------------------------------------------------------------------

HIV+ 53% (47%) 48% (42%) 42% (37%) 33% (27%) 33% (26%) 31% (24%)

HIV + --- --- --- 25% (18%) 25% (17%) 24% (17%)

diagnosed

HIV- 33% (26%) 33% (27%) 32% (26%) 27% (21%) 29% (22%) 30% (23%)

-----------------------------------------------------------------------------------------------------------------------------------------------------

**Table 8.** Origin of new infections for one example epidemic, based on using modal values for parameters. This shows the proportion of new infections that have been acquired from a person in primary HIV infection by year, and the proportion of new infections that have been acquired from a long term partner by year. For infections from people with primary infection, there are little data from sub-Saharan Africa to our knowledge. Data from men who have sex with men indicate that around one third of new infections may come from people who are themselves in primary infection (13). For proportion of people infected by a long term partner, compare with ref 14.

Status of source partner

Primary infection Long term partner

1990 2000 2010 1990 2000 2010

-----------------------------------------------------------------------------------------------------------------------------

Males 30% 17% 24% 40% 51% 46%

Females 30% 25% 27% 69% 76% 71%

-----------------------------------------------------------------------------------------------------------------------------

**Table 9.** HIV prevalence by gender and age using modal values for parameters.

Males Females

1985

-------------------------------------------------------------------------------------------------------------------------------------------------

Age group

15- 5.0 7.4

25- 8.0 8.3

35- 5.3 4.5

45- 3.0 1.7

55- 1.4 0.7

1990

-------------------------------------------------------------------------------------------------------------------------------------------------

Age group

15- 6.3 10.3

25- 15.0 18.7

35- 11.4 12.6

45- 5.9 5.1

55- 2.8 1.7

1995

-------------------------------------------------------------------------------------------------------------------------------------------------

Age group

15- 6.5 11.6

25- 19.1 27.7

35- 18.0 23.2

45- 9.2 10.3

55- 4.0 3.5

-------------------------------------------------------------------------------------------------------------------------------------------------

2000

-------------------------------------------------------------------------------------------------------------------------------------------------

Age group

15- 5.3 9.8

25- 18.5 28.7

35- 20.9 30.0

45- 11.5 14.8

55- 4.8 5.0

-------------------------------------------------------------------------------------------------------------------------------------------------

2005

-------------------------------------------------------------------------------------------------------------------------------------------------

Age group

15- 4.0 8.0

25- 17.0 27.3

35- 22.6 34.0

45- 14.5 19.9

55- 5.9 6.9

-------------------------------------------------------------------------------------------------------------------------------------------------

3. Natural history of HIV infection

The model of the natural history of HIV and the effect of antiretroviral therapy has been derived previously and validated (see refs 15, 16 and associated supplementary material). Below we set out the structure of the model and explain what parameters represent. There is some uncertainty associated with many of the values (see parameter distributions at end of this document).

**3. 1. Determination of changes in viral load and CD4 count**

**Initial log_10_ viral load** (V_set_) is sampled from Normal(4.0,0.5^2^)

This viral load (V_set_) is assumed to be that reached after primary infection. It is not used to determine the risk of transmission in primary infection itself.

**Initial CD4 count**, modelled on the square root scale, is partially dependent on initial viral load and given by

Square root CD4 count = *mean_sqrtcd4_inf* - (2 x V_set_) + Normal(0,2^2^)

(Distribution of *mean_sqrtcd4_inf:* Normal(30,2^2^))

Initial virus is assumed to be R5-tropic. Shift to presence of X4 virus is assumed to depend on viral load. Probability of a shift per 3 months is given by 10^v^ x 0.0000004, where v is the current log_10_ viral load.

Viral load change (vc) from period t-1 to period t (i.e. in 3 months) is given by sampling from a Normal distribution N ( *gx* x 0.02275, 0.05^2^)*.* (distribution of gx*:* lognormal( ln1.0, 0.20^2^))

CD4 count changes from period t-1 to t are dependent on the current viral load (i.e. viral load at time t-1) and are given by sampling from a Normal distribution with standard deviation *sd_cd4* and mean *fx* (distribution: lognormal( ln0.8, 0.20^2^)) times the values as follows

Viral load Change in

at t-1 square root

CD4 count

(per 3 mths)

-----------------------------------------------

< 3.0 -0.03

3-0 -0.08

3.5- -0.15

4.0- -0.20

4.5- -0.50

5.0- -1.00

5.5- -2.00

6.0- -2.50

-----------------------------------------------

The change additionally is affected by the current age as follows:

Age Additional change in

square root CD4 count

----------------------------------------------------------------------

< 20 +0.15

20- +0.09

25- +0.06

30- +0.0

35- +0.0

40- -0.06

45- -0.09

50- -0.15

60- -0.20

----------------------------------------------------------------------

People with X4 virus present experience an additional change in square root CD4 count of -0.25.

These estimates are derived based on synthesis of evidence from natural history studies (17-25) and were selected in conjunction with other relevant parameter values to provide a good fit to the incubation period distribution. Differences that have been found in initial viral load by sex, age and risk group are not currently incorporated in the model.

**Table 10.** Incubation period by age. Kaplan-Meier percent with WHO 4 Event. Using modal values for parameters. Compare with ref 26.

Age at infection Years from infection

1 3 5 10 15 20

------------------------------------------------------------------------------------------------

15- 0.3% 3% 10% 43% 75% 91%

25- 0.8% 5% 15% 55% 84% 95%

35- 0.9% 9% 22% 64% 91% 98%

45- 1.4% 11% 26% 74% 93% 99%

55- 1.8% 11% 30% 75% 96% 100%

------------------------------------------------------------------------------------------------

4. HIV testing and diagnosis of HIV infection

Date of start of testing for HIV (initially only in those with WHO 4 conditions (date_start_testing): 1996

Rate of increase in testing probability over time (this is testing that is independent of presence of WHO stage 3 or 4 conditions; i.e. VCT) (test_increase_rate) = lognormal( ln0.006,1.5^2^)

Testing is assumed to occur with a frequency no more than annual.

For people who have had no condomless sex partners since last test the probability of testing is reduced 10-fold.

Initial test probability for those with WHO 4 condition (this increases by 0.008 per 3 mths after testing is introduced, up to 2015) (test_rate_who4): lognormal( ln0.2,0.3^2^)

Initial test probability for those with TB (this increases by 0.005 per 3 mths after testing is introduced, up to 2015) (test_rate_tb): lognormal( ln0.1,0.3^2^)

Initial test probability for those with current WHO 3 condition (this increases by 0.0012 per 3 mths after testing is introduced, up to 2015) (test_rate_who3): lognormal( ln0.03,0.3^2^)

Probability that person is hard to reach for testing (with reduced probability of testing, unless with WHO 4 condition, in which case they will be tested) (rate_hardreached): lognormal( ln0.3, 0.30^2^)

Probability that person is not possible to reach for testing (with zero probability of testing, unless with WHO 4 condition, in which case they will be tested) (rate_noreached): lognormal( ln0.1, 0.30^2^)

5. Use of ART

**Initiation of ART**

In the base model ART initiation in diagnosed people before 2010 is determined by a CD4 count < 200 or the development of a WHO 4 event. After 2010 this is determined by a CD4 count < 350.

**Interruption of ART**

The basic rate of interruption due to patient choice is *rate_int_choice* (distribution: lognormal( ln0.05, 1.00)) - this rate is greater with current toxicity (2-fold) and greater in patients with a greater tendency to be non-adherent (1.5-fold if adherence average 0.6 – 0.79 and 2-fold if adherence average < 0.60).

if adherence average > 0.8 then 30% chance that interruption coincides with interrupting/stopping visits to the clinic, if 0.6 <= adhav < 0.8 then 45% chance, if adhav < 0.6 then 60% chance.

The rate of interruption due to choice is likely to vary by setting. The above rates were derived to be consistent with data from mainly European and US cohorts (27-30).

The basic rate of interruption due to interruption of the drug supply is *prob_supply_interrupted per 3 mnths* (distribution: lognormal( ln0.01, 0.30^2^)).

Again, the distribution of values considered is indicated at the end of this document.

**Interruption of ART without clinic/clinician being aware**

It is known that in some instances people on ART have such poor adherence that they have in fact interrupted or stopped ART entirely but, in the same way that the clinician is not always aware of the true adherence level, they are also not always aware when the person has completely interrupted ART. This means that the clinician may think a patient is virologically failing, because viral load is high, when in fact this is due to interruption rather than resistance. This can be seen from studies on people with virologic failure in which a proportion have no identified resistance mutations (31-33). Thus, when a person interrupts ART (but remains under care) we introduce a variable that indicates whether the clinician is unaware. *clinic_not_aw_int_frac* distribution: beta(5,5). If a patient has interrupted ART with the clinician unaware then not only is the patient (wrongly) classified (by the clinician) as virologically failing, but a switch to second line can occur.

**Re-initiation of ART after interrupting in patients still under follow-up**

For patients who have interrupted ART due to choice but are still under clinic follow-up, the probability of restarting ART per 3 months in the base model is *prob_restart* (distribution: lognormal( ln0.2,0.50)) This probability is increased 3-fold if a new WHO 3 condition has occurred at t-1, and 5-fold if a new WHO 4 condition has occurred at t-1.

This was derived from consideration of estimates of the proportion of people who had started ART who were on ART (e.g. 30). This will likewise vary by setting and is investigated in sensitivity analyses.

For patients who have interrupted ART due to interruption of supply the probability of restarting ART per 3 months in the base model is *prob_supply_resumed* (distribution: lognormal( ln0.8, 0.30^2^))

**Switch to second line after failure of first line ART**

Whatever the criterion for the need to switch to second line ART is determined, the probability of switching per 3 month period after the criterion is met is *pr_switch*_line (distribution: beta(3,9)). (See refs 34-35)

**Loss to follow-up while off ART**

The probability per 3 months of interrupting/stopping clinic visits (i.e. being lost to follow-up) is *rate_lost* (distribution: lognormal( ln0.05, 0.4^2^)) if adherence average > 0.8. This is increased by 1.5 fold if 0.6 < adherence average < 0.8 and by 2-fold if adherence average < 0.6. Concept of adherence average is described below.

For people lost to follow-up who are asymptomatic, the probability of returning to clinic per 3 months is *rate_return* (distribution: lognormal( ln0.05,0.30^2^)) if adherence average > 0.8. This is decreased by 2-fold if 0.6 < adherence average < 0.8 and by 3-fold if adherence average < 0.6. If a person develops a new WHO 3 or 4 event then they are assumed to return to the clinic with probability 1. These will vary by setting (36-39).

**Adherence pattern**

There are two components to the adherence. Each patient has a fixed “tendency to adhere” but their actual adherence varies from period to period, both at random and according to the presence of symptoms. Adherence is measured on a scale of 0 to 1.

Component which is fixed over time for a given patient

Adherence average (*adhav*) is a measure of the patient’s tendency to adhere, a fixed value for a patient, with a certain period-to-period variability (adhvar). Adherence at any one period is determined as follows (although with modifications explained below):- adh(t) = *adhav* + Normal(0,*adva*r^2^)

There are various adherence pattern distributions (numbered 1-5) considered.

Adherence pattern 1

3% probability *adhav* = 0.50 *adhvar* = 0.2

3% probability *adhav* = 0.80 *adhvar* = 0.2

14% probability *adhav* = 0.90 *adhvar*  = 0.06

80% probability *adhav* = 0.95 *adhvar* = 0.05

Adherence pattern 2

5% probability *adhav* = 0.50 *adhvar* = 0.2

10% probability *adhav* = 0.80 *adhvar* = 0.2

27% probability *adhav* = 0.90 *adhvar*  = 0.06

38% probability *adhav* = 0.90 *adhvar* = 0.05

20% probability *adhav* = 0.95 *adhvar* = 0.05

Adherence pattern 3

15% probability *adhav* = 0.50 *adhvar* = 0.2

15% probability *adhav* = 0.70 *adhvar* = 0.2

50% probability *adhav* = 0.90 *adhvar*  = 0.06

20% probability *adhav* = 0.95 *adhvar* = 0.05

Adherence pattern 4

30% probability *adhav* = 0.50 *adhvar* = 0.2

30% probability *adhav* = 0.70 *adhvar* = 0.2

10% probability *adhav* = 0.90 *adhvar*  = 0.06

30% probability *adhav* = 0.95 *adhvar* = 0.05

Adherence pattern 5

30% probability *adhav* = 0.50 *adhvar* = 0.2

30% probability *adhav* = 0.60 *adhvar* = 0.2

10% probability *adhav* = 0.70 *adhvar*  = 0.06

30% probability *adhav* = 0.90 *adhvar* = 0.05

if adh(t) > 1 then adh(t)=1

(Distribution of adherence patterns: 1 5%, 2 20%, 3 15%, 4 30%, 5 30%).

We originally have used adherence pattern 2. These estimates are based partially on observed adherence data (40, 41), but also on adherence levels required to produce observed estimates of rates of resistance development and virologic failure and also data on the proportion of patients at first virologic failure who have no resistance mutations present (31-33,42). It is clear from such data in more recent years that the great majority of patients who started ART with 3 or more drugs are sufficiently adherent that virologic failure rates (and so resistance accumulation is likely to have been slow also) are low (34, 43-44).

Since we wished to consider a range of epidemics and programmes, some for which the average adherence pattern may be poor, and since we wished to consider epidemics for which levels of transmitted drug resistance are high, we also considered the poorer adherence profiles 3-5. To enable full consideration of the effect of adherence we also included adherence pattern 1, which is better adherence than we consider has generally been observed in any treated popultion.

**Effective adherence**

We also considered the concept of effective adherence, which reflects predicted adequacy of drug levels, whereby for those on regimens that do not include an NNRTI the effective adherence is as the adherence, but for those on NNRTI-containing regimens the effective adherence is the adherence + *add_eff_adh_nnrti* (distribution: lognormal( ln0.1, 0.30^2^)), reflecting the long half life of these drugs (46) . Additionally, it is assumed that patients on ART are susceptible to occasional (rate 0.02 per year) severe temporary drops in drug level (i.e. effective adherence level), leaving them susceptible to viral rebound (but with low risk of resistance as the effective adherence drop is so profound). This phenomenon is assumed to be 3 times more frequent among those on protease inhibitor regimens. This latter assumption is the only plausible means (at least within our model framework) to explain why virologic failure occurring on boosted protease inhibitor regimens often occurs in the absence of resistance.

**Effect of viral load measurement above 1000 cps/mL on adherence**

Studies have indicated that viral load frequently returns to undetectable after a measured value > 1000 cps/mL, largely attributable to targetting of adherence support. Adherence is assumed to be incremented by an average *adh_effect_of_vm_pop* (by an amount that varies by individual) when the viral load has been measured to be above 1000 copies/mL in the past 6 month period.

6. Effect of ART on viral load, CD4 count, resistance development and drug toxicity

Patients on ART - Determination of viral load, CD4 count, acquisition of new resistance mutations between t-1 and t (variable “newmut(t)”) (Table 10)

These depend on the effective adherence between t-1 and t, number of active drugs (nactive(t-1)), time on the current regimen and the current viral load itself. The way the values are generated is detailed on the following pages. For those on NNRTI regimens the new mutations risk is assumed to be that for the effective adherence category of 0.5 – 0.8 (i.e. maximal) even if the effective adherence is below 0.5, reflecting the fact that NNRTI resistance develops easily, even when drug exposure is very low.

The changes in viral load and CD4 count are based on observed data and observational studies (and to some extent randomized trials, although responses tend to be better in trial participants), and provide long term estimates of virologic failure rates and CD4 count increases in ART which are broadly consistent with observed. Values of the “new mutation risk” (*new_mut*) parameter have been chosen in conjunction with the translation of presence of mutations into reduce drug activity to provide estimates of resistance accumulation consistent with those observed in clinical practice (45,47-54)

**Table 10a. Viral load (mean change from viral load max), CD4 count change (mean change between t-1 and t), and new mutation risk in first 3 months.** For 0 active drugs, these are the changes regardless of time from start of regimen. For viral load this is the mean of a Normal distribution with standard deviation 0.2, from which the patient's value/change is sampled. For the CD4 count patients vary in their underlying propensity for CD4 rise on ART (given by sampling from lognormal(1,0.5^2^) and the CD4 count change given here is multiplied by this factor. For the new mutation risk, this is a number that is multiplied by the viral load (mean of values at t-1 and t). The resulting number ("newmut") is used when assessing whether a new mutation or mutations have arisen (see below).

Effective Number of active drugs

adherence

between

t-1 & t 3 2.75 2.5 2.25 2.0 1.75 1.5 1.25 1 0.75 0.5 0.25 0

-------------------------------------------------------------------------------------------------------------------------------------------------------------------------------------------------------------

Viral load > 0.8 -3.0 -2.6 -2.2 -1.8 -1.5 -1.25 -0.9 -0.8 -0.7 -0.55 -0.4 -0.3 -0.3

(log change > 0.5, < 0.8 -2.0 -1.6 -1.2 -1.1 -0.9 -0.8 -0.6 -0.5 -0.4 -0.25 -0.1 -0.05 -0.1

from vmax) < 0.5 -0.5 -0.4 -0.3 -0.25 -0.2 -0.15 -0.0 +0.05 +0.1 +0.1 +0.1 +0.1 -0.0

CD4 count > 0.8 +50 +45 +40 +35 +30 +25 +20 +17 +13 +10 +5 -2 -15

change > 0.5, < 0.8 +30 +30 +23 +20 +15 +13 +10 +8 +5 +3 +0 -7 -17

(t-1 to t) < 0.5 +5 +4 +3 +2 +1 -1 -3 -6 -10 -11 -12 -13 -18

new mutation > 0.8 0.002 0.01 0.03 0.05 0.1 0.15 0.2 0.3 0.4 0.45 0.5 0.5 0.5

risk > 0.5, < 0.8 0.15 0.15 0.2 0.25 0.3 0.3 0.3 0.35 0.4 0.45 0.5 0.5 0.5

(x log viral load) < 0.5 0.05 0.05 0.05 0.05 0.05 0.05 0.05 0.05 0.05 0.05 0.05 0.05 0.05

-------------------------------------------------------------------------------------------------------------------------------------------------------------------------------------------------------------

**Table 10b.** Summary of viral load (mean absolute value or mean change from viral load max) between 3-6 months, and after 6 months if viral load at t-1 > 4 logs. This is the mean of a Normal distribution with standard deviation 0.2, from which the patient's value/change is sampled.

Effective adherence Effective adherence Number of active drugs

between between

t-2 & t-1 t-1 & t 3 2.75 2.5 2.25 2.0 1.75 1.5 1.25 1 0.75 0.5 0.25

------------------------------------------------------------------------------------------------------------------------------------------------------------------------------------------------------------

> 0.8 > 0.8 0.5 0.8 1.2 1.4 2.0 2.7 -1.7 -1.15 -0.9 -0.75 -0.6 -0.4

> 0.5, < 0.8 > 0.8 1.2 1.2 1.2 1.4 -2.0 -1.6 -1.2 -1.05 -0.9 -0.7 -0.5 -0.35

< 0.5 > 0.8 1.2 1.2 1.2 1.4 -2.0 -1.6 -1.2 -1.0 -0.9 -0.7 -0.5 -0.2

> 0.8 > 0.5, < 0.8 1.2 1.6 1.8 2.2 2.4 -2.4 -1.5 -0.9 -0.7 -0.55 -0.4 -0.3

> 0.5, < 0.8 > 0.5, < 0.8 2.5 2.5 2.5 2.5 -1.2 -1.1 -0.8 -0.65 -0.5 -0.35 -0.2 -0.05

< 0.5 > 0.5, < 0.8 -2.0 -1.8 -1.5 -1.35 -1.2 -1.1 -0.8 -0.65 -0.5 -0.2 -0.2 -0.05

> 0.8 < 0.5 -0.5 -0.4 -0.3 -0.25 -0.2 -0.15 -0.10 -0.05 +0.0 +0.0 +0.0 +0.0

> 0.5, < 0.8 < 0.5 -0.5 -0.4 -0.3 -0.25 -0.2 -0.15 -0.10 -0.05 +0.0 +0.0 +0.0 +0.0

< 0.5 < 0.5 -0.5 -0.4 -0.3 -0.25 -0.2 -0.15 -0.10 -0.05 +0.0 +0.0 +0.0 +0.0

------------------------------------------------------------------------------------------------------------------------------------------------------------------------------------------------------------

**Table 10c.** Summary of CD4 count change (mean change between t-1 and t) between 3-6 months, and after 6 months if viral load at t-1 > 4 logs. For the CD4 count patients vary in their underlying propensity for CD4 rise on ART (given by sampling from lognormal(1,0.5^2^) and the CD4 count change given here is multiplied by this factor. Once the mean of the underlying CD4 count is obtained, to obtain the (underlying) CD4 count, variability (SD = 1.2) is added on the square root scale

Effective adherence Effective adherence Number of active drugs

between between

t-2 & t-1 t-1 & t 3 2.75 2.5 2.25 2.0 1.75 1.5 1.25 1 0.75 0.5 0.25

------------------------------------------------------------------------------------------------------------------------------------------------------------------------------------------------------------

> 0.8 > 0.8 +30 +28 +25 +23 +21 +19 +3 -5 -9 -10.5 -12 -14

> 0.5, < 0.8 > 0.8 +30 +28 +25 +23 +7.5 +1.5 -4.5 -7 -9 -11 -13 -14.5

< 0.5 > 0.8 +30 +28 +25 +23 +7.5 +1.5 -4.5 -7.5 -9 -11 -13 -16

> 0.8 > 0.5, < 0.8 +15 +13 +10 +8 +7 +13.5 +0 -9 -11 -12.5 -14 -15

> 0.5, < 0.8 0.5, < 0.8 +15 +13 +10 +8 -4.5 -6 -10 -11.5 -13 -14.5 -16 -17.5

< 0.5 > 0.5, < 0.8 +7.5 +4.5 +0 -2 -4.5 -6 -10 -11.5 -13 -16 -16 -17.5

> 0.8 < 0.5 -13 -14 -15 -15.5 -16 -1 -17 -17.5 -18 -18 -18 -18

> 0.5, < 0.8 < 0.5 -13 -14 -15 -15.5 -16 -16.5 -17 -17.5 -18 -18 -18 -18

< 0.5 < 0.5 -13 -14 -15 -15.5 -16 -16.5 -17 -17.5 -18 -18 -18 -18

-------------------------------------------------------------------------------------------------------------------------------------------------------------------------------------------------------------

**Table 10d. Summary of new mutation risk between 3-6 months, and after 6 months if viral load at t-1 > 4 logs.** This is a number that is multiplied by the viral load (mean of values at t-1 and t). The resulting number ("newmut") is used when assessing whether a new mutation or mutations have arisen (below).

Effective adherence Effective adherence Number of active drugs

between between

t-2 & t-1 t-1 & t 3 2.75 2.5 2.25 2.0 1.75 1.5 1.25 1 0.75 0.5 0.25

---------------------------------------------------------------------------------------------------------------------------------------------------------------------------------------------------------

> 0.8 > 0.8 0.002 0.01 0.03 0.05 0.05 0.1 0.2 0.3 0.4 0.45 0.5 0.5

> 0.5, < 0.8 > 0.8 0.002 0.01 0.03 0.05 0.05 0.1 0.2 0.3 0.4 0.45 0.5 0.5

< 0.5 > 0.8 0.05 0.05 0.03 0.05 0.05 0.1 0.2 0.3 0.4 0.45 0.5 0.25

> 0.8 > 0.5, < 0.8 0.10 0.15 0.2 0.2 0.3 0.3 0.3 0.35 0.4 0.45 0.5 0.5

> 0.5, < 0.8 > 0.5, < 0.8 0.10 0.15 0.2 0.2 0.3 0.3 0.3 0.35 0.4 0.45 0.5 0.5

< 0.5 > 0.5, < 0.8 0.10 0.15 0.2 0.2 0.3 0.3 0.3 0.35 0.4 0.45 0.5 0.25

> 0.8 < 0.5 0.05 0.05 0.05 0.05 0.05 0.05 0.05 0.05 0.05 0.05 0.05 0.05

> 0.5, < 0.8 < 0.5 0.05 0.05 0.05 0.05 0.05 0.05 0.05 0.05 0.05 0.05 0.05 0.05

< 0.5 < 0.5 0.05 0.05 0.05 0.05 0.05 0.05 0.05 0.05 0.05 0.05 0.05 0.05

-----------------------------------------------------------------------------------------------------------------------------------------------------------------------------------------------------------

**Table 10e.** Summary of viral load (mean change from viral load max), CD4 count change (mean change between t-1 and t), and new mutation risk after 6 months, where viral load at t-1 < 4 logs. For viral load this is the mean of a Normal distribution with standard deviation 0.2, from which the patient's value/change is sampled. For the CD4 count patients vary in their underlying propensity for CD4 rise on ART (given by sampling from lognormal(1,0.5^2^) and the CD4 count change given here is multiplied by this factor. For the new mutation number, this is a number that is multiplied by the viral load (mean of values at t-1 and t). The resulting probability ("newmut") is used when assessing whether a new mutation or mutations have arisen (see below).

Effective Number of active drugs

adherence

between

t-1 & t 3 2.75 2.5 2.25 2.0 1.75 1.5 1.25 1 0.75 0.5 0.25

-------------------------------------------------------------------------------------------------------------------------------------------------------------------------------------------------

Viral load > 0.8 0.5 0.9 1.2 1.6 -2.5 -2.0 -1.4 -1.15 -0.9 -0.75 -0.6 -0.3

(absolute value > 0.5, < 0.8 1.2 1.2 1.2 1.4 -1.2 -1.0 -0.7 -0.6 -0.5 -0.4 -0.3 -0.1

or log change < 0.5 -0.5 -0.4 -0.3 -0.25 -0.2 -0.2 -0.1 -0.1 -0.1 -0.1 -0.1 -0.0

from vmax)

CD4 count > 0.8 +30 +28 +25 +23 +21 +19 +3 -5 -9 -10.5 -12 -12

change > 0.5, < 0.8 +15 +13 +10 +8 -4.5 -7.5 -10 -12 -13 -14 -15 -15

(t-1 to t) < 0.5 -13 -14 -15 -15.5 -16 -16.5 -17 -17 -18 -17 -17 -17

new mutation > 0.8 0.002 0.01 0.03 0.08 0.10 0.15 0.2 0.3 0.4 0.45 0.5 0.5

(x viral load) > 0.5, < 0.8 0.15 0.18 0.2 0.25 0.3 0.3 0.3 0.35 0.4 0.45 0.5 0.5

< 0.5 0.05 0.05 0.05 0.05 0.05 0.05 0.05 0.05 0.05 0.05 0.05 0.05

-------------------------------------------------------------------------------------------------------------------------------------------------------------------------------------------------

**Variable patient-specific tendency for CD4 count rise on ART**

There is variability in the tendency for the CD4 count to rise on ART, for a given level of viral load suppression. For scenarios in the above table in which the CD4 count change is positive the CD4 count change is modified by this patient-specific factor (i.e. it is fixed for each patient), which is given by sampling for each patient from

Exp ( N(0, (*sd_patient_cd4_rise_art*)^2^)

(Distribution of sd_patient_cd4_rise_art): Normal(0.5,1^2^))

**Reduced CD4 count rise for faster CD4 count risers after 2 continuously years on ART**

To reflect the fact that the rate of CD4 count increase on ART tends to diminish with time (51), for those with patient-specific factor determining the CD4 count rise on ART > 1, this factor is modified by a factor 0.25 after 2 years of continuous treatment.

**Accelerated rate of CD4 count loss if PI not present in regimen**

The rate of change in CD4 count in people on failing regimens is largely based on data from the PLATO collaboration, for which patients were mainly on regimens containing a PI. If the regimen does not contain a PI the change in CD4 count per 3 months is modified (in the base model) by *poorer_cd4_rise_on_failing_nnrti (distribution*: Normal(-6,3^2^) cells/mm^3^. This applies regardless of viral load level, so PIs are assumed to lead to a more beneficial CD4 count change than NNRTIs (56).

**Variability in individual (underlying) CD4 counts for people on ART**

Once the mean of the underlying CD4 count is obtained as described above for people on ART, to obtain the CD4 count, variability (*sd_cd4* distribution: Normal(1.2,0.2^2^)) is added on the square root scale. The estimate was based on unpublished analyses.

**Viral load and CD4 count changes during ART interruption**

Viral load returns to previous maximum viral load (vmax) in 3 months and adopts natural history changes thereafter.

CD4 rate of decline returns to natural history changes (ie those in ART naïve patients) after 9 months, unless the count remains > 200 above the CD4 nadir

Rate of CD4 count decline depends on current viral load. c(t) is the CD4 count at time t, cmin(t) is the CD4 count nadir measured by time t and cc(t-1) is the change in CD4 count from t-1 to t. v

if time off ART = 3 or if time off ART > 3 months and CD4 in previous period is > 300 above the minimum CD4 count to date

v(t) = vmax(t-1)

if v(t) > 5 then cc(t-1) = Normal (-200,10^2^)

if 4.5 <= v(t) < 5 then cc(t-1) = Normal (-160,10^2^)

if v(t) < 4.5 then cc(t-1) = Normal (-120,10^2^)

If this leads to c(t) < cmin(t) (CD4 nadir) then c(t) is set to cmin(t)

if time off ART = 6 months:-

if v(t) > 5 then cc(t-1) = Normal (-100,10^2^)

if 4.5 <= v(t) < 5 then cc(t-1) = Normal (-90,10^2^)

if v(t) < 4.5 then cc(t-1) = Normal (-80,10^2^)

if time off ART = 9 months:-

if v(t) > 5 then cc(t-1) = Normal (-80,10^2^)

if 4.5 <= v(t) < 5 then cc(t-1) = Normal (-70,10^2^)

if v(t) < 4.5 then cc(t-1) = Normal (-60,10^2^)

This is broadly based on evidence from a number of analyses of the effects of ART interruption (27-29,57-65).

**Incidence of new current toxicity and continuation of existing toxicity**

Toxicities including gastrointestinal symptoms, rash, hepatoxicity, CNS toxicity, lipodystrophy, hypersensitvity reaction, peripheral neuropathy and nephrolithiasis can occur with certain probability on certain specific drugs. These probabilities are based broadly on evidence from trials and cohort studies, although there are no common definitions for some conditions which complicates this.

**Table 11.**

Drug Toxicity Risk of development Probability of continuation

per 3 months if pre-existing

--------------------------------------------------------------------------------------------------------------------------------------

lopinavir nausea 0.1 (1.5-fold higher in 1st year) 0.5

ddI

zidovudine

lopinavir diarrhoea 0.03 (1.5-fold higher in 1^st^ year) 0.2

ddI 0.05 0.2

efavirenz rash 0.03 (this is a one-off risk in 1^st^ 3 mths)

nevirapine 0.1 (ditto)

efavirenz CNS 0.1 (in 1^st^ year, 0 after) 0.8 (in 1^st^ year)

0.9 (after 1 year)

d4T lipodystrophy 0.05 1.0

zdv 0.015 1.0

d4T peripheral 0.02 (1.5 fold higher in 1^st^ year) 1.0 (if remain on d4T)

ddI neuropathy 0.01 (1.5 fold higher in 1^st^ year) 1.0 (if remain on ddI)

nevirapine acute hepatitis 0.05 (one off risk in first and 2^nd^ 3 month

periods)

zidovudine anaemia 0.03 (1.5 fold higher in 1^st^ year) 0.2

zidovudine headache 0.1 (1.5 fold higher in 1^st^ year) 0.4

ddI pancreatitis 0.001

d4T 0.001

zidovudine lactic acidosis 0.001

ddI

d4T

--------------------------------------------------------------------------------------------------------------------------------------

**Switching of drugs due to toxicity**

If toxicity is present then individual drugs may be switched due to toxicity. In most cases, the switch is to another in the same class, if such a drug (that has not been previously failed nor stopped due to toxicity) is available. This will vary by setting and availability of alternative drugs.

**First line ART failure definition**

The default definition for first line failure depends on availability of CD4 count and viral load measures and is as follows:

CD4 / Vl Measures available Failure definition

---------------------------------------------------------------------------------------------------------------------------------

None New WHO stage 4 condition

Two separate new WHO stage 3 conditions

CD4 counts CD4 count < 100 /mm3 after >1.5 years on continuous ART*

CD4 count < 200 /mm3 after > 3 years on continuous ART*

(CD4 count measured 6 monthly)

or clinical failure as defined above

Viral load viral load > 1000 cps/mL*

(Viral load measured at 6 months and then every 12 months)

---------------------------------------------------------------------------------------------------------------------------------

* confirmation is required with a subsequent value also

**Table 12**. Kaplan-Meier estimates of percent with viral load failure (> 500 after > 6 months on ART), resistance (predicted susceptibility < 50%) to at least one drug, CD4 count rise of > 200/mm^3^, using modal values for parameter distributions (but with adherence pattern 2, the one thought to be most realistic), assuming no ART interruption and restricting to people with no transmitted drug resistance and assuming no super-infection with resistant virus. Compare, for example, with refs 34, 37, 66.

Years from start of ART

1 3 5 10 20

--------------------------------------------------------------------------------------------------------------------------------------------

Viral load failure 6% 12% 16% 23% 33%

Resistance 10% 16% 19% 28% 40%

CD4 count rise of 28% 65% 79% 89% 98%

> 200/mm^3^

--------------------------------------------------------------------------------------------------------------------------------------------

**Table 13**. Kaplan-Meier estimates of percent interrupting ART, percent lost to follow up after starting ART, and percent dead, using modal values of parameter distributions (see end of this document, but with adherence pattern 2, the one thought to be most realistic). Both restricting to those in care and including those lost to care.

Years from start of ART

1 3 5 10 20

---------------------------------------------------------------------------------------------------------------------------------------------

Interruption of ART 28% 55% 72% 91% 99%

Loss to care 11% 25% 35% 59% 85%

Death (in those under care) 7% 14% 18% 33% 58%

Death (incl in those lost) 7% 17% 25% 49% 79%

---------------------------------------------------------------------------------------------------------------------------------------------

**Table 14.** Cumulative risk of death and returning to care after first being lost to follow up after starting ART using modal values of parameter distributions (see end of this document, but with adherence pattern 2, the one thought to be most realistic). Competing risk approach.

Years from first lost

(after starting ART)

1 3 5

-----------------------------------------------------------------------------------------------------------------------

Death while lost 18% 33% 37%

Return after loss to care 28% 51% 59%

----------------------------------------------------------------------------------------------------------------------

7. Emergence of specific resistance mutations and their effect on drug activity

**Accumulation of resistance mutations**

*newmut* (see Table 10 above) is a probability used to indicate the level of risk of new mutations arising in a given 3 month period. If this chance comes up in a given 3 month period (determined by sampling from the binomial distribution) then the following criteria operate.

**Table 15.**

Drug on Mutation Probability of arising (given newmut=1)

-------------------------------------------------------------------------------------------------------------------------

3TC M184V 0.80

d4t or new TAM if not on 3TC

zidovudine increase by 1: 0.20

increase by 2: 0.01

if on 3TC

increase by 1: 0.12

increase by 2: 0.01

ddI L74V 0.01

ddI or d4t 65R if on zidovudine

0.01

If not on zidovudine

0.04

ddI or d4t or Q151M 0.02

zidovudine

nevirapine NNRTI mutation 0.80

efavirenz

lopinavir/r 32 0.04

47 0.04

82 0.04

-------------------------------------------------------------------------------------------------------------------------

These values are chosen, in conjunction with values of *newmut*, to provide estimates of accumulation of specific classes of mutation consistent with those observed in clinical practice (45, 67, 68). They reflect a greater propensity for some mutations to arise than others. This probably relates to the ability of the virus to replicate without the mutations (e.g. probably very low in the presence of 3TC for virus without M184V) as well as the replicative capacity of virus with the mutations. Over time as more data accumulate it may be possible improve these estimates of rates of accumulation of specific mutations.

**New resistance to NNRTI arising as a result of ART interruption**

It is assumed that due to the long half life of NNRTIs nevirapine and efavirenz, stopping of a regimen containing one of these drugs is associated with a probability *risk_res_stopping_nn* (distribution: lognormal( ln0.05 ,0.30^2^)) of an NNRTI resistance mutation arising (see, for example, ref 69).

**Loss of acquired mutations from majority virus**

It is assumed that mutations tend to be lost from majority virus with a certain probability from 3 months after stopping to take a drug that selects for that mutation. The probability of losing mutations per 3 months (from 3 months after stopping) is as follows (ref 70-76).

**Table 16.**

----------------------------------------

M184V 0.8

L74V 0.6

Q151M 0.6

K65R 0.6

TAMS (lose all) 0.4

NNRTI mutations 0.2

Protease mutations 0.2

---------------------------------------

Mutations are regained in majority virus if a drug selecting for the mutation is again started.

**Determination of level of resistance to each drug**

**Table 17.**

Mutation Drug Level of resistance (1 = full resistance)

---------------------------------------------------------------------------------------------------------------------

M184V 3TC 0.75

1-2 TAMS zidovudine or d4t 0.5

(no 3TC in regimen)

3-4 TAMS zidovudine or d4t 0.75

(no 3TC in regimen)

5-6 TAMS zidovudine or d4t 1.00

(no 3TC in regimen)

1-2 TAMS zidovudine or d4t 0.5

(3TC in regimen

- no M184V ever)

3-4 TAMS zidovudine or d4t 0.75

(3TC in regimen

- no M184V ever)

5-6 TAMS zidovudine or d4t 0.75

(3TC in regimen

- no M184V ever)

1-2 TAMS zidovudine or d4t 0.25

(3TC in regimen

- M184V ever)

3-4 TAMS zidovudine or d4t 0.5

(3TC in regimen

- M184V ever)

5-6 TAMS zidovudine or d4t 0.75

(3TC in regimen

- M184V ever)

Q151M zidovudine or d4t 0.75

K65R d4t 0.5

> 3 TAMS ddI 0.5

L74V ddI 0.75

K65R ddI 0.75

Q151M ddI 0.75

NNRTI mutation nevirapine or 1.00

efavirenz

1 from Pr 32,47,82 lopinavir/r 0.25

2 from Pr 32,47,82 lopinavir/r 0.5

3 from Pr 32,47,82 lopinavir/r 0.75

4 from Pr 46, 82, 84, 90 lopinavir/r max(r_lpr, 0.5)

2 or 3 from Pr 46, 82, 84, 90 lopinavir/r max(r_lpr, 0.25)

-------------------------------------------------------------------------------------

These rules approximately follow the interpretation systems for conversion of mutations present on genotypic resistance test into a predicted level of drug activity (or, equivalently, of resistance; e.g. 77-79). Currently interpretation systems differ to some degree in their prediction of activity for some drugs.

**Calculation of activity level of each drug**

This is given by 1-level of resistance. For lopinavir/r (in base model) it is given by 2 – (2 x level of resistance); i.e. assumed higher potency. Activity levels of each drug in the regimen are summed to give the total number of active drugs.

8. Risk of clinical disease and death in HIV infected people

**Occurrence of WHO 4 diseases**

(see ref 56, 80-81)

Rate of WHO 4 diseases according to CD4 count per 3 months

If cd4 > 650 rate=0.002

if 500 < cd4 < 650 rate=0.010 if 450 < cd4 < 500 rate=0.013

if 400 < cd4 < 450 rate=0.016 if 375 < cd4 < 400 rate=0.020

if 350 < cd4 < 375 rate=0.022 if 325 < cd4 < 350 rate=0.025

if 300 < cd4 < 325 rate=0.030 if 275 < cd4 < 300 rate=0.037

if 250 < cd4 < 275 rate=0.045 if 225 < cd4 < 250 rate=0.055

if 200 < cd4 < 225 rate=0.065 if 175 < cd4 < 200 rate=0.080

if 150 < cd4 < 175 rate=0.10 if 125 < cd4 < 150 rate=0.13

if 100 < cd4 < 125 rate=0.17 if 90 < cd4 < 100 rate=0.20

if 80 < cd4 < 90 rate=0.23 if 70 < cd4 < 80 rate=0.28

if 60 < cd4 < 70 rate=0.32 if 50 < cd4 < 60 rate=0.40

if 40 < cd4 < 50 rate=0.50 if 30 < cd4 < 40 rate=0.80

if 20 < cd4 < 30 rate=1.10 if 10 < cd4 < 20 rate=1.80

if 0 < cd4 < 10 rate=2.50

Independent effect of viral load

if v < 3 rate = rate x 0.2

if 3 <= v < 4 rate = rate x 0.3

if 4 <= v < 4.5 rate = rate x 0.6

if 4.5 <= v < 5 rate = rate x 0.9

if 5 <= v < 5.5 rate = rate x 1.2

if 5.5 <= v rate = rate x 1.6

**Independent effect of age**

rate = rate x (age / 38)^1.2^

**Independent effect of PJP prophylaxis**

If patient on PJP prophylaxis then this rate is multiplied by 0.8.

If CD4 count is meausured and current value < 350 /mm3 then patient assumed to have 80% chance of starting PJP prophylaxis after 1996

If patient has current WHO stage 3 or 4 condition they are assumed to have an 80% chance of starting PJP prophylaxis

If CD4 count is measured then PJP prophylaxis assumed to stop if current value > 350/mm3.

If the patient has been continuously on ART for 2 years with no WHO 3 or 4 condition in previous 6 months then it is assumed that PJP prophylaxis is stopped.

**Independent effect of being on ART**

For patients on a single drug regimen this risk is multiplied by 0.9, for patients on a two drug regimen it is multiplied by 0.85 and for patients on a 3 drug regimen it is multiplied by 0.8, to reflect that being on HAART has a positive effect on risk of AIDS and death independent of latest CD4 count and viral load.

**Occurrence of WHO 3 diseases**

As for WHO 4 except risk is fold_incr_who3 higher (distribution fold_incr_who3 higher: lognormal( ln5,0.30^2^)).

**Risk of HIV-related death**

As for WHO 4 except risk *fold_decr_hivdeath*-fold lower (distribution *fold_decr_hivdeath*: lognormal(ln0.25, 0.30^2^)).

CD4-, viral load- age-specific death rate raised *incr_death_rate_tb*-fold (distribution: lognormal( ln2, 0.30^2^)) if current TB and *incr_death_rate_adc*-fold (distribution: lognormal( ln5,0.50^2^)) if current WHO 4 disease. Assume 15% of HIV-related deaths (ie not including deaths that arise due to background mortality rates) are classified as non-HIV-related.

**References**

1. Kuller LH, et al. Cigarette smoking and mortality. Preventive Medicine. 1991; 20:638-654.
2. Johnson LF, et al. Sexual behaviour patterns in South Africa and their association with the spread of HIV: Insights from a mathematical model. Demographic Research 2009; 21:289-340.
3. Gregson S et al. Methods to reduce social desirability bias in sex surveys in low-development settings - Experience in Zimbabwe. Sexually Transmitted Diseases 2002; 29: 568-575.
4. Shisana O, Rehle T, Simbayi LC, Parker W, Zuma K, Bhana A, Connolly C, Jooste S, Pillay V et al. (2005) South African National HIV Prevalence, HIV Incidence, Behaviour and Communication Survey, 2005. Cape Town: HSRC Press.
5. Shisana O, Rehle T, Simbayi LC, Zuma K, Jooste S, Pillay-van-Wyk V, Mbelle N, Van Zyl J, Parker W, Zungu NP, Pezi S & the SABSSM III Implementation Team (2009) *South African national HIV prevalence, incidence, behaviour and communication survey 2008:A turning tide among teenagers?* Cape Town: HSRC Press).
6. Hollingsworth TD, Anderson RM, Fraser C. HIV-1 transmission, by stage of infection. J Infect Dis 2008; 198:687–93
7. Nicolosia A. The efficiency of male-to-female and female-to-male sexual transmission of the human-immunodeficiency-virus -a study of 730 stabke couples. Epidemiology 1994; 5 : 570 1994
8. Yang RR, Gui X, Benoit JL, et al. [The Comparison of Human Immunodeficiency Virus Type 1 Transmission between Couples through Blood or Sex in Central China](http://apps.isiknowledge.com.libproxy.ucl.ac.uk/full_record.do?product=WOS&search_mode=GeneralSearch&qid=9&SID=2C9C5dfP8j@D1749IN9&page=1&doc=6). Japanese J Infect Dis 2010; 63:283-285.
9. Cohen MS. Sexually transmitted diseases enhance HIV transmission: no longer a hypothesis
   Lancet 1998; 351 : 5.
10. Corvasce et al. Evidence of differential selection of HIV-1 variants carrying drug-resistant mutations in seroconverters. Antiviral Therapy 2006; 11:329 -334.
11. Turner et al. Diminished Representation of HIV-1 Variants Containing Select Drug Resistance–Conferring mutations in Primary HIV-1 Infection. JAIDS 2004; 37: 1627-1631)
12. <http://www.doh.gov.za/aids/docs/sum-report.html>
13. Pao D et al. Transmission of HIV-1 during primary infection: relationship to sexual risk and sexually transmitted infections. AIDS 19 : 85 2005
14. Dunkle KL, et al. New heterosexually transmitted HIV infections in married or cohabiting couples in urban Zambia and Rwanda: an analysis of survey and clinical data. Lancet 2008; 371:2183-2191.
15. Phillips AN, Pillay D, Miners A, Bennett D, Gilks CF, Lundgren JD. Outcomes from monitoring of patients on antiretroviral therapy in resource-limited settings with viral load, CD4 cell count, or clinical observation alone: a computer simulation model. Lancet 2008; 371: 1443–51.
16. Phillips AN, Pillay D, Garnett G, Bennett D, Vitoria M, Cambiano V, Lundgren JD. Effect on transmission of HIV-1 resistance of timing of implementation of viral load monitoring to determine switches from first to second line antiretroviral regimens in resource-limited settings. AIDS 2011; 25:843-850.
17. Pantazis N, Touloumi G. Bivariate modelling of longitudinal measurements of two human immunodeficiency type 1 disease progression markers in the presence of informative drop-outs. JRSS C 2005; 54: 405-423.
18. Sabin CA, Devereux H, Phillips AN, et al. Course of viral load throughout HIV-1 infection. JAIDS 2000; 23:172-177.
19. Hubert J-B, Burgard M, Dussaix E, et al. Natural history of serum HIV-1 RNA levels in 330 patients with known date of infection. AIDS 2000; 14:123-131.
20. O'Brien TR, Rosenberg PS, Yellin F, et al. Longitudinal HIV-1 RNA levels in a cohort of homosexual men. JAIDS 1998; 18:155-161.
21. Henrard DR, Phillips JF, Muenz LR et al. Natural history of HIV-1 cell-free viraemia. JAMA 1995; 274: 554-558.
22. Lyles RH, Munoz A, Yamashita TE, et al. Natural history of human immunodeficiency virus type 1 viraemia after seroconversion and proximal to AIDS in a large cohort of homosexual men. J Infect Dis 2000; 181 (3): 872-880.
23. Touloumi G, Pantazis N, Babiker AG, et al. Differences in HIV RNA levels before the initiation of antiretroviral therapy among 1864 individuals with known HIV-1 seroconversion dates. AIDS 2004; 18 (12): 1697-1705.
24. Mellors JW, Munoz A, Giorgi JV, et al. Plasma viral load and CD4(+) lymphocytes as prognostic markers of HIV-1 infection. Ann Intern Med 1997; 126 (12): 946-954.
25. Koot M, Keet IPM, Vos AHV, et al. Prognostic value of human HIV-1 biological phenotype for the rate of CD4+ cell depletion and progression to AIDS. Ann Intern Med 1993; 118: 681-688.
26. Darby SC, Ewart DW, Giangrande PLF, et al. Importance of age at infection with HIV-1 for survival and development of AIDS in UK haemophilia population. Lancet 1996; 347: 1573–79.
27. d'Arminio Monforte A, Cozzi Lepri A, Phillips AN, et al. Interruption of HAART in HIV clinical practice. Results from the ICONA study. JAIDS 2005; 38: 407-416.
28. Li X, Margolick JB, Conover CS, et al. Interruption and discontinuation of HART in the MACS. JAIDS 2005; 38: 3:320-328.
29. Mocroft A, Youle M, Moore A, et al. Reasons for modification and discontinuation of antiretrovirals: results from a single treatment centre. AIDS 2001; 15 (2): 185-194.
30. Cambiano V, Lampe FC, Rodger AJ, Smith CJ, Geretti AM, Lodwick RK, Puradiredja DI, Johnson M, Swaden L, Phillips AN. Long-term trends in adherence to antiretroviral therapy from start of HAART. AIDS  2010; 24:1153-1162.
31. Hoffmann C, et al. Viremia, Resuppression, and Time to Resistance in Human Immunodeficiency Virus (HIV) Subtype C during First-Line Antiretroviral Therapy in South Africa Clin Infect Dis 2009; 49:1928–35
32. Wallis CL, et al. Varied Patterns of HIV-1 Drug Resistance on Failing First-Line Antiretroviral Therapy in South Africa. JAIDS 2010;53:480–484
33. Hamers RL, et al. Patterns of HIV-1 Drug Resistance After First-Line Antiretroviral Therapy (ART) Failurein 6 Sub-Saharan African Countries: Implications for Second-Line ART Strategies. Clin Infect Dis 2012;54(11):1660–9.
34. Fox MP et al. Rates and Predictors of Failure of First-line Antiretroviral Therapy and Switch to Second-line ART in South Africa JAIDS 2012; 60:428–437
35. Johnston V et al. Outcomes Following Virological Failure and Predictors of Switching to Second-line Antiretroviral Therapy in a South African Treatment Program. JAIDS 2012; 61:370–380
36. Charurat M, Oyegunle M, Benjamin R, et al.Patient Retention and Adherence to Antiretrovirals in a Large Antiretroviral Therapy Program in Nigeria: A Longitudinal Analysis for Risk Factors. PLOS One 2010; 5:Article Number: e10584
37. Anaky MF, Duvignac J, Wemin L, et al. Scaling up antiretroviral therapy for HIV-infected children in Cote d'Ivoire: determinants of survival and loss to programme. Bull World Health Org 2010;  88: 490-499
38. Larson BA, Brennan A, McNamara L, et al. Early loss to follow up after enrolment in pre-ART care at a large public clinic in Johannesburg, South Africa. Trop Med & Int Health 2010; 15: 43-47.
39. Fox MP, Rosen S. Patient retention in antiretroviral therapy programs up to three years on treatment in sub-Saharan Africa, 2007-2009: systematic review. Trop Med & Int Health  2010; 15:1-15.
40. Mills EJ, Nachega JB, Buchan I, Orbinski J, Attaran A, Singh S et al. Adherence to antiretroviral therapy in sub-Saharan Africa and North America - A meta-analysis. JAMA 2006; 296 (6): 679-690.
41. Bangsberg DR, Moss AR, Deeks SG et al. Paradoxes of adherence and drug resistance to HIV antiretroviral therapy. J Antimicrob Chem 2004; 53 (5): 696-699.
42. Mackie N, Phillips AN, Kaye S, et al. Antiretroviral drug resistance in HIV-1 infected patients with low-level viraemia. J Infect Dis 2010; 201:1303-1307
43. Lampe FC, Smith CJ, Madge S, Kinloch-de Loes S, Tyrer M, Sabin CA, Chaloner C, Youle M, Johnson MA, Phillips AN. Success of HIV clinical care according to demographic group among sexually-infected patients in a routine clinic population, 1999 to 2004. Arch Int Med 2007;167:692-700
44. El-Khatib Z, Ekstrom AM, Ledwaba J, et al.Viremia and drug resistance among HIV-1 patients on antiretroviral treatment: a cross-sectional study in Soweto, South Africa. AIDS  2010; 24:1679-1687.
45. Johannessen A, Naman E, Kivuyo SL, et al. Virological efficacy and emergence of drug resistance in adults on antiretroviral treatment in rural Tanzania. BMC Iinf Dis 2009; 9:108.
46. Cheeseman SH, et al. [Pharmacokinetics of nevirapine – initial single rinsing dose study in humans](http://apps.isiknowledge.com.libproxy.ucl.ac.uk/full_record.do?product=WOS&search_mode=GeneralSearch&qid=41&SID=2C9C5dfP8j@D1749IN9&page=4&doc=37). Antimicrobial Agenets and Chem 1993; 37: 178-182.
47. UK HIV Drug Resistance Database and UK CHIC. Long term probability of detection of HIV-1 drug resistance after starting antiretroviral therapy in routine clinical practice. AIDS 2005; 19 (5): 487-494.
48. Harrigan PR, Hogg RS, Dong WWY, et al. Predictors of HIV drug resistance mutations in a large antiretroviral naïve cohort initiating triple antiretroviral therapy. J Infect Dis 2005; 191: 339:347.
49. Ledergerber B, Egger M, Opravil M, et al. Clinical progression and virologic failure on HAART in HIV-1 patients: a prospective cohort study. Lancet 1999; 353:863-868.
50. Phillips AN, Staszewski S, Weber R, et al. HIV viral load response to antiretroviral therapy according to the baseline CD4 cell count and viral load. JAMA 2001; 286:2560-2567.
51. Staszewski S, Miller V, Sabin C, et al. Virological response to protease inhibitor therapy in an HIV clinic cohort AIDS 1999; 13 (3): 367-373.
52. Staszewski S, Miller V, Sabin C, et al. Determinants of sustainable CD4 lymphocyte count increases in response to antiretroviral therapy. 1999; AIDS 13 (8): 951-956.
53. Gallant J, Staszewski S, Pozniak A, et al. Efficacy and safety of Tenofovir DF vs stavudine in combination therapy in antiretroviral-naïve patients. JAMA 2004; 292: 191-201.
54. van Leth F, Phanuphak P, Ruxrungtham K, et al. Comparison of first-line antiretroviral therapy with regimens including nevirapine, efavirenz, or both drugs, plus stavudine and lamivudine: a randomized open-label trial, the 2NN study. Lancet 2004; 363: 1253-1263.
55. Mocroft A, Phillips A, Gatell J, et al. Normalisation of CD4 counts in patients with HIV-1 infection and maximum virological suppression who are taking combination antiretroviral therapy: an observational cohort study. Lancet 2007; 370:407-413.
56. The PLATO Collaboration. Predictors of trend in CD4-positive T-cell count and mortality among HIV-1-infected individuals with virological failure to all three antiretroviral-drug classes. Lancet 2004; 364(9428)**:** 51-62.
57. Youle M, Janossy G, Turnbull W, et al. Changes in CD4 lymphocyte counts after interruption of therapy in patients with viral failure on a protease inhibitor containing regimen. AIDS 2000; 14:1717-1720.
58. Skiest DJ, Morrow P, Allen B, et al. Is it safe to stop antiretroviral therapy in patients with preantiretroviral CD4 cell counts > 250 cells/ul. JAIDS 2004; 37: 1351-1357.
59. Lawrence J, Mayers D, Huppler Hullsiek K, et al. Structured treatment interruption in patients with multidrug resistant HIV. N Engl J Med 2003; 349:837-846.
60. Tebas P, Henry K, Mondy K et al. Effect of prolonged discontinuation of successful antiretroviral therapy on CD4+ T cell decline in HIV infected patients: implications for intermittent therapeutic strategies. J Infect Dis 2002; 186:851-854
61. Fischer M, Hafner R, Schneider C, et al. HIV RNA in plasma rebounds within days during structured treatment interruption. AIDS 2003; 17:195-199.
62. Boschi A, Tinelli C, Ortolani P, et al. CD4+ cell-guided treatment interruptions in chronic HIV-infected patients with good response to HAART. AIDS 2004; 18:2381-2389.
63. Achenbach CJ, Till M, Palella FJ, et al. Extended antiretroviral treatment interruption in HIV infected patients with long term suppression of plasma HIV RNA. HIV Medicine 2005; 6:7-12.
64. Thiebaut R, Pellegrin I, Chene G, et al. Immunological markers after long term treatment interruption in chronically HIV-1 infected patients with CD4 cell count above 400 x 10^6^ cells/l. AIDS 2005; 19:53-61.
65. Wit FWNM, Blanckenberg DH, Brinkman K, et al. Safety of long-term interruption of successful antiretroviral therapy: the ATHENA cohort study. AIDS 2005; 19: 345-348.
66. Cozzi Lepri A, et al. Long term probability of detecting HIV drug resistance in drug-naïve patients starting non nucleoside reverse transcriptase inhibitor- or ritonavir boosted protease inhibitor- containing antiretroviral therapy. Clin Infect Dis 2010.
67. Harrigan PR, Hogg RS, Dong WWY, et al. Predictors of HIV drug resistance mutations in a large antiretroviral naïve cohort initiating triple antiretroviral therapy. J Infect Dis 2005; 191: 339:347.
68. Sigaloff K, et a. Accumulation of HIV Drug Resistance Mutations in Patients Failing First-Line Antiretroviral Treatment in South Africa. AIDS Res Hum Retr 2012; 28:171-175.
69. Fox Z, Phillips AN, Cohen C, et al. Viral resuppression and detection of drug resistance following interruption of a suppressive non-nucleoside reverse transcriptase inhibitor-based regimen. AIDS 2008; 22:2279-2289.
70. Devereux HL, Youle M, Johnson MA, et al Rapid decline in detectability of HIV-1 drug resistance mutations after stopping therapy. AIDS  1999; 13:F123-F127.
71. Devereux HL, Emery VC, Johnson MA, et al. Replicative fitness in vivo of HIV-1 variants with multiple drug resistance associated mutations. J Med Virol 2001:; 65:218-224.
72. Deeks SG, Grant RM, Wrin T, et al. Persistence of drug-resistant HIV-1 after a structured treatment interruption and its impact on treatment response. AIDS 2003; 17:361-370.
73. Birk M, Svedhem V, Sonnerborg A. Kinetics of HIV-1 RNA and resistance-associated mutations after cessation of antiretroviral combination therapy. AIDS 2001; 15:1359-1368.
74. Walter H, Low P, Harrer T, et al. No evidence for persistence of multidrug resistant viral strains after a 7-month treatment interruption in an HIV-1 infected individual. JAIDS 2002; 31:137-146.
75. Hance AJ, Lemiale V, Izopet J, et al. Changes in HIV-1 populations after treatment interruption in patients failing antiretroviral therapy. J Virol 2001; 75:6410-6417.
76. Tarwater PM, Parish M, Gallant JE. Prolonged treatment interruption after immunologic response to HAART. Clin Infect Dis 2003; 37:1541-1548.
77. <http://www.rega.kuleuven.be/cev/index.php?id=30>
78. <http://hivdb.stanford.edu/pages/algs/HIVdb.html>
79. <http://www.hivfrenchresistance.org/>
80. Phillips A, CASCADE Collaboration. Short-term risk of AIDS according to current CD4 cell count and viral load in antiretroviral drug-naïve individuals and those treated in the monotherapy era. *AIDS*, 2004. 18(1):51-8.
81. Phillips AN, Lee CA, Elford J, et al. More rapid progression to AIDS in older HIV-infected people: the role of CD4+ T-cell counts. JAIDS 1991; 4:970-975.

**Parameters and distributions for uncertainty analysis and value used for main analysis**

**Sexual behaviour**

Sexual behaviour model structure: 80% base structure, 20% alternative structure (with no sex workers – see details) (*main analysis: base structure)*

Change in propensity to have a long term condomless sex partner (“risk”) after HIV diagnosis (ch_risk_diag): Beta (10,5) (*main analysis: 0.67)*

Change in propensity to have short term (“new”) condomless sex partners after HIV diagnosis (ch_risk_diag_newp): Beta(6,2) = round(ch_risk_diag_newp,0.001) (*main analysis: 0.75 )*

Threshold of HIV prevalence at which population level change in condomless sex behaviour changes (prev_threshold_rb_change): Uniform(0.1,0.4) (*main analysis:0.1)*

Change in propensity to have condomless sex (“risk behaviour”) with short term partners after threshold for population level change in condomless sex behaviour reached (ch_risk_beh_newp): Beta(6,6) (*main analysis:0.5)*

Change in propensity to have a long term condomless sex (“risk behaviour”) with short term partners after threshold for population level change in condomless sex behaviour reached (ch_risk_beh_ep): Beta(7,5) (*main analysis:0.6)*

Rate of starting new long term condomless sex partnership in 15-25 year age group (eprate): lognormal(ln0.1,0.25^2^) (*main analysis:0.1)*

Poisson mean for moderately high short term partner group (see model details) (highsa): uniform(2.5,6.5)) (*main analysis:4.5)*

Poisson mean for highest short term partner group (see model details) (swn): gamma(4,8) (*main analysis: 7)*

Factor to change overall average level of condomless sex with short term partners. This is sampled to be inversely correlated with highsa and swn to ensure that a high proportion of epidemics generate have a prevalence in the 3-25% range by 2015 (without this factor we would generate many more epidemics where prevalence is very low, if highsa, swn and newp_factor are all low, or very high, if all three values are high – the aim is to achieve good sampling efficiency without imposing too much constraint on epidemics generated) (newp_factor)

For base sexual beheviour model structure: base value: lognormal( ln((5.5/highsa)x(25/swn)),0.1^2^)

For alternative sexual behaviour model structure: lognormal( ln(6.0/highsa),0.1^2^)

(*main analysis:4.5)*

Proportion of the population who have a lifetime reduced number of condomless sex partners (see model details) (p_rred_p): uniform(0.1,0.4) (*main analysis:0.1)*

**Transmission**

Fold difference in transmission rate for a given viral load (see Model details for base assumption on transmission rate by viral load (fold_tr): lognormal( ln1.0,0.3^2^) (*main analysis:1.0)*

Rate of transmission in primary HIV infection (lasting 3 months) (tr_rate_primary): beta(25,80) (*main analysis:0.24)*

Transmission rate when plasma viral load is < 500 cps/mL (tr_rate_undetec_vl): min(1, lognormal( ln0.0001,3^2^)) (*main analysis: 0.0001)*

Fold higher rate of transmission from women to men, compared with men to wormen (fold_change_w): lognormal(ln1.5,0.3^2^) (*main analysis:1.5)*

Fold higher rate of transmission in young women compared with older women (fold_change_yw): lognormal( ln2,0.5^2^) (*main analysis:2)*

Fold higher rate of transmission if current STI present (fold_change_sti): lognormal( ln3,0.3^2^) (*main analysis:3)*

Fold lower transmission rate for short term partners compared with long term (reflecting average lower number of sex acts) (fold_tr_newp): beta(6,10) (*main analysis:0.38)*

Super-infection (with consequent risk of acquiring new mutations) occurs: yes 90% chance, no 10% chance (*main analysis:yes)*

Adjustment to factor determining extent to which some transmitted resistance is effectively immediately lost (even from minority virus) (res_trans_factor) lognormal(1.0 , 0.3^2^) (*main analysis:1.00)*

Probability per 3 months of loss of persistence of transmitetd mutations from majority virus to minority virus (same for each mutation) (rate_loss_persistence): lognormal( ln0.04,0.3^2^) (*main analysis:0.04)*

Date of initiation of PMTCT = 1998

Probability per 3 months of NNRTI resistance emergence in women due to single dose nevirapine for MTCT (note, actual use of PMTCT is not modelled, only the risk of resistance as a result) (rate_nnres_pmtct): lognormal (ln0.005, 0.60^2^) (*main analysis:0.005)*

Probability per 3 months of loss of NNRTI mutations, acquired due to PMTCT, from majority virus to become only in minority virus (rate_loss_nnres_pmtct_maj): lognormal( ln0.25, 0.3^2^) (*main analysis:0.25)*

Probability per 3 months of loss of virus with NNRTI mutations acquired due to PMTCT, from minority virus to effectively be extinct altogether (rate_loss_nnres_pmtct_min): lognormal( ln0.25, 0.3^2^) ) (*main analysis:0.25)*

Probability of male circumcision per 3 months: (rate_circ): lognormal( ln0.001, 0.5^2^) ) (*main analysis:0.001)*

Effect of male circumcision: reduced rate of acquisition = 0.5 fold

**HIV testing**

Rate of increase in testing probability over time (test_increase_rate) = lognormal( ln0.006,1.5^2^) ) (*main analysis:0.008)*

Maximum frequency of testing: annual (people do not have tests more often than annually) ) (*main analysis:annually)*

Date of start of testing for HIV (initially only in those with WHO 4 conditions (date_start_testing): 1996

Initial test probability for those with WHO condition (this increases by 0.008 per 3 mths after testing is introduced, up to 2015) (test_rate_who4): lognormal( ln0.2,0.3^2^) ) (*main analysis:0.20)*

Initial test probability for those with TB (this increases by 0.005 per 3 mths after testing is introduced, up to 2015) (test_rate_tb): lognormal( ln0.1,0.3^2^) ) (*main analysis:0.10)*

Initial test probability for those with current WHO 3 condition (this increases by 0.0012 per 3 mths after testing is introduced, up to 2015) (test_rate_who3): lognormal( ln0.03,0.3^2^) ) (*main analysis:0.03)*

Probability that person is hard to reach for testing (with reduced probability of testing, unless with WHO 4 condition, in which case they will be tested) (rate_hardreached): lognormal( ln0.3, 0.30^2^) ) (*main analysis:0.3)*

Probability that person is not possible to reach for testing (with zero probability of testing, unless with WHO 4 condition, in which case they will be tested) (rate_noreached): lognormal( ln0.1, 0.30^2^) ) (*main analysis:01)*

**Natural progression**

Probability of being lost (unlinked to care) at diagnosis (prob_loss_at_diag): beta(10,20) ) (*main analysis:0.33)*

Initial CD4 count at infection (square root scale) (mean_sqrtcd4_inf): Normal(30,2^2^) (*main analysis:30)*

Decrease per year in mean initial CD4 count at infection: 0 (*main analysis:0)*

Factor adjusting basic rate of natural cd4 decline (see model details) (fx): lognormal( ln1.0, 0.20^2^) ) (*main analysis:0.8)*

Factor adjusting basic rate of natural viral load change (see model details) (gx): lognormal( ln1.0, 0.20^2^) ) (*main analysis:1.00)*

Fold increase in risk of WHO 3 condition, compared with risk of WHO 4 condition, for given level of CD4 count, viral load and age (fold_incr_who3): lognormal( ln5,0.30^2^) ) (*main analysis:5)*

Fold decrease in risk of HIV-related death, compared with risk of WHO 4 condition, for given level of CD4 count, viral load and age (fold_decr_hivdeath): lognormal(ln0.25, 0.30^2^) ) (*main analysis:0.25)*

Fold difference in risk of WHO 4 condition, for given level of CD4 count, viral load and age, compared with base assumption (see model details) (fold_change_in_risk_base_rate): lognormal( ln1.0, 0.30^2^) ) (*main analysis:1.00)*

Increase in death rate in 3 months period in which a WHO 4 condition is present (incr_death_rate_adc): lognormal(ln5,0.50^2^) ) (*main analysis:5)*

Increase in death rate in 3 months period in which TB is present incr_death_rate_tb): lognormal( ln2, 0.30^2^) ) (*main analysis:2)*

Fold difference in non HIV related mortality, compared with base assumption (fold_change_ac_death_rate): lognormal(ln1, 0.25^2^) ) (*main analysis:1)*

**HIV monitoring, loss, return, interruption of art and restarting**

Risk of loss to follow-up per 3 mths among those not on ART (rate_lost): lognormal( ln0.05, 0.4^2^) ) (*main analysis:0.05)*

Probability of simultaneously being lost to follow-up amongst those stopping ART (prob_lost_art): beta(4,9) ) (*main analysis:0.31)*

Probability (per 3 mths) of return to care for person lost (if no WHO 4 condition present – value is 1 if present) (rate_return):lognormal (ln0.05,0.30^2^) ) (*main analysis:0.2)*

Basic probability of restart of ART in those remaining under care who have stopped/interrupted ART (this is also influenced by presence of WHO 3 or 4 conditions) (rate_restart): lognormal( ln0.2,0.50^2^) ) (*main analysis:0.2)*

**ART**

ART introduction date: 2003

Probability of switching to second line treatment, given first line failure (by whatever definition is being used) (pr_switch_line): beta(3,9) (*main analysis:0.25)* )

Pattern of adherence (see model details for description of distribution of adherence levels for each adherence pattern – higher adherence pattern numbers reflect poorer adherence): 2 50%, 3 50%. ) (*main analysis: 2)*

Reduction in adherence resulting from presence of TB or a WHO 4 condition (red_adh_tb_adc): lognormal (ln0.1, 0.5^2^) ) (*main analysis:0.1)*

Average reduction in adherence resuting from current toxicity (the actual reduction varies by inidvidual person) (red_adh_tox_pop): lognormal( ln0.05, 0.5^2^) ) (*main analysis:0.05)*

Additional "effective" adherence for people on NNRTI regimens due to longer half life (add_eff_adh_nnrti): lognormal(ln0.1, 0.30^2^) (*main analysis:0.1* )

Population averge effect of recent viral load measurement of high viral load on adherence (amount differs by individual) (adh_effect_of_vm_pop): lognormal( ln0.1, 0.5^2^) ) (*main analysis:0.05)*

Average change in adherence on second line (degree of change varies by indvidual – note this can be a positive or negative change) (altered_adh_sec_line_pop) = Normal(0.05, 0.05^2^) ) (*main analysis:0.05)*

Extent to which the CD4 change is more favourable on a virologically failing BPI-regimen compared with an NNRTI-regimen (poorer_cd4_rise_on_failing_nnrti): Normal(-6,3^2^) (*main analysis: -6)*

Standard deviation for intra-subject variation in CD4 count (sd_cd4): Normal(1.2,0,2^2^) (*main analysis:1.2)*

Standard deviation for the measurement error in CD4 count (sd_measured_cd4): Normal(2.0,0,2^2^) (*main analysis:2.0)*

Base probability of interrupting ART per 3 mths (actual probability also depends on time on continuous ART, presence of current toxicity and average adherence – see model details) (rate_int_choice): lognormal( ln0.02, 0.35^2^) ) (*main analysis:0.02)*

Probability of drug stock out, and hence ART initerrupted (prob_supply_interrupted): lognormal( ln0.005, 0.30^2^) ) (*main analysis:0.005)*

Probability that drug supply resumed during stock-out (prob_supply_resumed): lognormal( ln0.8, 0.30^2^) ) (*main analysis:0.8)*

Fold difference in risk of mutations arising, for given number of active drugs, viral load and current adherence level, compared with base risk (see model details) (fold_change_mut_risk): lognormal( ln1 , 0.30^2^) ) (*main analysis:1.0)*

Similarly, specifically for thymidine analogue mutations: (fold_change_tams_risk): lognormal( ln1, 0.30^2^) ) (*main analysis:1.0)*

Similarly, specifically for Q151M cross nucleoside resistance mutation: (fold_change_151_risk): lognormal( ln1, 0.30^2^) ) (*main analysis:1.0)*

Standard deviation representing inter-patient variation in rate of CD4 rise - when CD4 is rising (sd_patient_cd4_rise_art): Normal(0.5,0.1^2^) (*main analysis:0.5)*

Risk of NNRTI resistance emergence due to stopping an NNRTI regimen (due to the tail in presence of drug meaning effective monotherapy) (risk_res_stopping_nn): lognormal( ln0.05, 0.30^2^) (*main analysis:0.05)*

Fraction of people who stop ART (and are still visiting the clinic) for whom the clinic is not aware of the interruption and is hence treating the patient as if they were on ART (and hence may switch to the next line having wrongly classified them as virologically failing): (clinic_not_aw_int_frac): beta(5,5) (*main analysis:0.5)*

**Table S1.** Mean over 2040-2060 of the following outcomes: HIV incidence (per 1000 person years), prevalence (%), % of whole population on ART (not only HIV infected), death rate (in whole population; per 100 person years), % of uninfected population age 15-65 with an on-going vaccine effect (i.e. vaccinated and up to date with boosters), 2040-2060, according to vaccination efficacy and implementation characteristics. 95% CI shown in grey.

**Difference from Vaccine efficacy**

**base scenario**

**Prevention 0% 30% 50% 90% 0% 0% 50% 90%**

**Viral load 0.0 0.0 0.0 0.0 1.0 2.0 1.0 2.0**

**(log_10_)**

--------------------------------------------------------------------------------------------------------------------------------------------------------------

Base scenario incidence 7.7 3.7 2.3 1.0 6.6 5.2 2.3 1.0

*7.5- 3.5- 2.2- 0.8- 6.2- 5.0- 2.1- 0.8-*

*7.9 3.9 2.4 1.2 6.8 5.4 2.5 1.2*

prevalence 13.5 9.5 7.7 5.6 12.9 12.0 7.8 5.7

*13.4 9.4- 7.5- 5.4- 12.8- 11.9- 7.7- 5.5-*

*13.6 9.6 7.9 5.8 13.0 12.1 7.9 5.9*

% on ART^+^ 8.6 6.6 5.6 4.4 7.6 6.6 5.4 4.3

*8.5- 6.5- 5.5- 4.3- 7.5- 6.5- 5.3- 4.2-*

*8.7 6.7 5.7 4.5 7.7 6.7 5.5 4.4*

death rate 1.19 1.06 1.01 0.93 1.09 1.03 0.98 0.92

*1.17- 1.04- 0.99- 0.91- 1.07- 1.01- 0.96- 0.90-*

*1.21 1.08 1.03 0.95 1.11 1.05 1.00 0.94*

% with ongoing 0% 45% 45% 45% 45% 45% 45% 45%

vaccine effect  *0%- 44%- 44%- 44%- 44%- 44%- 44%- 44%-*

*0% 46% 46% 46% 46% 46% 46% 46%*

Boosting to age 30 incidence 7.7 4.4 3.1 1.7 6.6 5.6 2.9 1.6

**7.6***- 4.2- 2.9- 1.5- 6.4- 5.4- 2.7- 1.4-*

*7.8 4.6 3.3 1.9 6.8 5.8 3.1 1.8*

prevalence 13.5 10.0 8.4 6.3 12.9 12.1 8.4 6.3

*13.4 9.8- 8.2- 6.1- 12.7- 11.9- 8.2- 6.1-*

*13.6 10.2 8.6 6.5 13.1 12.3 8.6 6.5*

% on ART^+^ 8.6 6.8 6.0 4.7 7.7 6.9 5.8 4.6

*8.5- 6.7- 5.9- 4.6- 7.6- 6.8- 5.7- 4.5-*

*8.7 6.9 6.1 4.8 7.8 7.0 5.9 4.7*

death rate 1.19 1.08 1.02 0.95 1.11 1.05 1.00 0.94

*1.17- 1.05- 0.99- 0.92- 1.08- 1.02- 0.97- 0.91-*

*1.21 1.11 1.05 0.98 1.14 1.08 1.03 0.97*

% with on-going 0% 26% 26% 26% 26% 26% 26% 26%

vaccine effect  *0%- 26%- 26%- 26%- 26%- 26%- 26%- 26%-*

*0% 26% 26% 26% 26% 26% 26% 26%*

50% booster completion rate incidence 7.7 4.3 3.0 1.5 6.7 5.6 2.8 1.5

*7.6- 4.1- 2.8- 1.3- 6.5- 5.4- 2.6- 1.3-*

*7.8 4.5 3.2 1.7 6.9 5.8 3.0 1.7*

prevalence 13.5 10.1 8.5 6.3 13.0 12.2 8.4 6.3

*13.4 9.9- 8.3- 6.1- 12.8- 12.0- 8.2- 6.1-*

*13.6 10.3 8.7 6.5 13.2 12.4 8.6 6.5*

% on ART^+^ 8.6 6.9 6.0 4.8 7.8 6.9 5.8 4.7

*8.5- 6.8- 5.9- 4.7- 7.7- 6.8- 5.7- 4.6-*

*8.7 7.0 6.1 4.9 7.9 7.0 5.9 4.8*

death rate 1.19 1.08 1.03 0.95 1.11 1.06 1.00 0.95

*1.17- 1.05- 1.00- 0.92- 1.08- 1.03- 0.97- 0.92-*

*1.21 1.11 1.06 0.98 1.14 1.09 1.03 0.98*

% with on-going 0% 34% 34% 34% 34% 34% 34% 34%

vaccine effect  *0%- 34%- 34%- 34%- 34%- 34%- 34%- 34%-*

*0% 34% 34% 34% 34% 34% 34% 34%*

25% booster completion rate incidence 7.7 4.9 3.7 2.1 7.1 6.1 3.6 2.1

*7.6- 4.7- 3.5- 1.9- 6.9- 5.9- 3.4- 1.7-*

*7.8 5.1 3.9 2.3 7.3 6.3 3.8 2.5*

prevalence 13.5 10.6 9.2 7.1 13.2 12.5 9.2 7.1

*13.3 10.4- 9.0- 6.9- 13.0- 12.3- 9.0- 6.9-*

*13.7 10.8 9.4 7.3 13.4 12.7 9.4 7.3*

% on ART^+^ 8.6 7.2 6.4 5.2 8.0 7.3 6.2 5.2

*8.5- 7.1- 6.3- 5.1- 7.9- 7.2- 6.1- 5.1-*

*8.7 7.3 6.5 5.3 8.1 7.4 6.3 5.3*

death rate 1.19 1.09 1.05 0.98 1.13 1.08 1.03 0.97

*1.17- 1.06- 1.02- 0.96- 1.11- 1.05- 1.00- 0.94-*

*1.21 1.12 1.07 1.00 1.15 1.11 1.06 1.00*

% with on-going 0% 23% 23% 23% 23% 23% 23% 23%

vaccine effect  *0%- 23%- 23%- 23%- 23%- 23%- 23%- 23%-*

*0% 23% 23% 23% 23% 23% 23% 23%*

40% coverage incidence 7.7 4.7 3.3 1.7 6.8 5.9 3.2 1.7

*7.6- 4.5- 3.1- 1.5- 6.6- 5.7- 3.0- 1.5-*

*7.8 4.9 3.5 1.9 7.0 6.1 3.4 1.9*

prevalence 13.5 10.4 8.8 6.5 13.1 12.4 8.9 6.5

*13.3 10.2- 8.6- 6.3- 12.9- 12.2- 8.7- 6.3-*

*13.7 10.6 9.0 6.7 13.3 12.6 9.1 6.7*

% on ART^+^ 8.6 7.0 6.2 4.9 7.9 7.1 6.0 4.8

*8.5- 6.9- 6.0- 4.7- 7.8- 7.0- 5.9- 4.6-*

*8.7 7.1 6.4 5.1 8.0 7.2 6.1 5.0*

death rate 1.19 1.09 1.04 0.96 1.12 1.07 1.02 0.95

*1.17- 1.06- 1.01- 0.93- 1.09- 1.04- 0.99- 0.92-*

*1.21 1.12 1.07 0.99 1.15 1.10 1.05 0.98*

% with on-going 0% 30% 30% 30% 30% 30% 30% 30%

vaccine effect  *0%- 30%- 30%- 30%- 30%- 30%- 30%- 30%-*

*0% 30% 30% 30% 30% 30% 30% 30%*

No adult catch-up program incidence 7.7 4.3 3.0 1.6 6.6 5.5 2.9 1.6

*7.6- 4.1- 2.8- 1.4- 6.4- 5.3- 2.7- 1.4-*

*7.8 4.5 3.2 1.8 6.8 5.7 3.1 1.8*

prevalence 13.5 10.5 9.0 7.2 13.0 12.2 9.0 7.2

*13.3 10.3- 8.8- 7.0- 12.8- 12.0- 8.8- 7.0-*

*13.7 10.7 9.2 7.4 13.2 12.4 9.2 7.4*

% on ART^+^ 8.6 7.2 6.5 5.4 7.8 7.0 6.2 5.4

*8.5- 7.0- 6.3- 5.2- 7.6- 6.8- 6.0- 5.2-*

*8.7 7.4 6.7 5.6 8.0 7.2 6.4 5.6*

death rate 1.19 1.10 1.06 1.00 1.12 1.07 1.04 0.99

*1.17- 1.07- 1.03- 0.97- 1.09- 1.04- 1.01- 0.96-*

*1.21 1.13 1.09 1.03 1.15 1.10 1.07 1.02*

% with on-going 0% 40% 40% 40% 40% 40% 40% 40%

vaccine effect  *0%- 40%- 40%- 40%- 40%- 40%- 40%- 40%-*

*0% 40% 40% 40% 40% 40% 40% 40%*

Tapering in effect over time incidence 7.7 6.6 5.9 4.6 6.5 5.2 5.0 3.5

*7.6- 6.4- 5.7- 4.4- 6.3- 5.0- 4.8- 3.3-*

*7.8 6.8 6.1 4.8 6.7 5.4 5.2 3.7*

prevalence 13.5 12.5 7.8 5.6 13.0 12.8 8.1 5.8

*13.3 12.3- 7.6- 5.4- 12.8- 12.6- 7.9- 5.6-*

*13.7 12.7 8.0 5.8 13.2 13.0 8.3 6.0*

% on ART^+^ 8.6 8.1 7.8 7.1 7.6 6.6 6.9 5.9

*8.5- 8.0- 7.7- 7.0- 7.5- 6.5- 6.8- 5.8-*

*8.7 8.2 7.9 7.2 7.7 6.7 7.0 6.0*

death rate 1.19 1.16 1.14 1.11 1.09 1.03 1.06 1.00

*1.17- 1.13- 1.11- 1.08- 1.06- 1.00- 1.03- 0.97-*

*1.21 1.19 1.17 1.14 1.12 1.06 1.09 1.03*

% with on-going 0% 45% 45% 45% 45% 45% 45% 45%

vaccine effect  *0%- 45%- 45%- 45%- 45%- 45%- 45%- 45%-*

*0% 45% 45% 45% 45% 45% 45% 45%*

Vaccination age 10 incidence 7.7 4.1 2.7 1.3 6.4 5.5 2.7 1.3

*7.6- 3.9- 2.5- 1.1- 6.2- 5.3- 2.5- 1.1-*

*7.8 4.3 2.9 1.5 6.6 5.7 2.9 1.5*

prevalence 13.5 10.0 8.4 6.3 12.9 12.2 8.5 6.3

*13.3 9.8- 8.2- 6.1- 12.7- 12.0- 8.3- 6.1-*

*13.7 10.2 8.6 6.5 13.1 12.4 8.7 6.5*

% on ART^+^ 8.6 6.9 6.0 4.8 7.7 6.9 5.8 4.7

*8.5- 6.8- 5.9- 4.7- 7.6- 6.8- 5.7- 4.6-*

*8.7 7.0 6.1 4.9 7.8 7.0 5.9 4.8*

death rate 1.19 1.08 1.03 0.96 1.10 1.05 1.01 0.95

*1.17- 1.05- 1.00- 0.93- 1.07- 1.02- 0.98- 0.92-*

*1.21 1.11 1.06 0.99 1.13 1.08 1.04 0.98*

% with on-going 0% 42% 42% 42% 42% 42% 42% 42%

vaccine effect  *0%- 42%- 42%- 42%- 42%- 42%- 42%- 42%-*

*0% 42% 42% 42% 42% 42% 42% 42%*

Rate vaccination 0.2/3mths incidence 7.7 3.9 2.5 1.1 6.3 5.3 2.4 1.1

*7.6- 3.7- 2.3- 0.9- 6.1- 5.1- 2.2- 0.9-*

*7.8 4.1 2.7 1.3 6.4 5.5 2.6 1.3*

prevalence 13.5 9.7 8.0 5.8 12.7 12.1 8.0 5.8

*13.3 9.5- 7.8- 5.6- 12.5- 11.9- 7.8- 5.6-*

*13.7 9.9 8.2 6.0 12.9 12.3 8.2 6.0*

% on ART^+^ 8.6 6.7 5.8 4.5 7.5 6.7 5.5 4.4

*8.5- 6.6- 5.7- 4.4- 7.4- 6.6- 5.4- 4.3-*

*8.7 6.8 5.9 4.6 7.6 6.8 5.6 4.5*

death rate 1.19 1.07 1.01 0.94 1.09 1.04 0.99 0.93

*1.17- 1.04- 0.98- 0.91- 1.06- 1.01- 0.96- 0.90-*

*1.21 1.10 1.04 0.97 1.12 1.07 1.02 0.96*

% with on-going 0% 43% 43% 43% 43% 43% 43% 43%

vaccine effect  *0%- 43%- 43%- 43%- 43%- 43%- 43%- 43%-*

*0% 43% 43% 43% 43% 43% 43% 43%*

Duration vaccine effect 2 incidence 7.7 3.9 2.4 1.1 6.4 5.3 2.4 1.1

years *7.6- 3.7- 2.2- 0.9- 6.2- 5.1- 2.2- 0.9-*

*7.8 4.1 2.6 1.3 6.6 5.5 2.6 1.3*

prevalence 13.5 9.7 7.9 5.8 12.7 12.1 8.1 5.8

*13.3 9.5 7.7- 5.6- 12.5- 11.9- 7.9- 5.6-*

*13.7 9.9 8.1 6.0 12.9 12.3 8.3 6.0*

% on ART^+^ 8.6 6.7 5.7 4.5 7.5 6.7 5.6 4.4

*8.5- 6.6- 5.6- 4.4- 7.4- 6.6- 5.5- 4.3-*

*8.7 6.8 5.8 4.6 7.6 6.8 5.7 4.5*

death rate 1.19 1.07 1.01 0.94 1.09 1.04 0.99 0.93

*1.17- 1.04- 0.98- 0.91- 1.06- 1.01- 0.96- 0.90-*

*1.21 1.10 1.04 0.97 1.12 1.07 1.02 0.96*

% with on-going 0% 44% 44% 44% 44% 44% 44% 44%

vaccine effect  *0%- 44%- 44%- 44%- 44%- 44%- 44%- 44%-*

*0% 44% 44% 44% 44% 44% 44% 44%*

Booster uptake decrease**incidence 7.7 4.0 2.7 1.3 6.5 5.4 2.6 1.3

*7.6- 3.8- 2.5- 1.1- 6.3- 5.2- 2.4- 1.1-*

*7.8 4.2 2.9 1.5 6.7 5.6 2.8 1.5*

prevalence 13.5 9.7 8.1 5.9 12.9 12.1 2.6 1.3

*13.3 9.5- 7.9- 5.7- 12.7- 11.9- 2.4- 1.1-*

*13.7 9.9 8.3 6.1 13.1 12.3 2.8 1.5*

% on ART^+^ 8.6 6.7 5.8 4.6 7.7 6.8 5.6 4.5

*8.5- 6.6- 5.7- 4.5- 7.6- 6.7- 5.5- 4.4-*

*8.7 6.8 5.9 4.7 7.8 6.9 5.7 4.6*

death rate 1.19 1.07 1.02 0.94 1.10 1.04 0.99 0.93

*1.17- 1.04- 0.99- 0.91- 1.07- 1.01- 0.96- 0.90-*

*1.21 1.10 1.05 0.97 1.13 1.07 1.02 0.96*

% with on-going 0% 35% 35% 35% 35% 35% 35% 35%

vaccine effect  *0%- 35%- 35%- 35%- 35%- 35%- 35%- 35%-*

*0% 35% 35% 35% 35% 35% 35% 35%*

Vaccine effect on VL in incidence 7.7 2.7 2.3 1.0 7.1 6.3 2.3 1.0

50% of people *7.6- 2.5- 2.1- 0.8- 6.9- 6.1- 2.1- 0.8-*

*7.8 2.9 2.5 1.2 7.3 6.5 2.5 1.2*

prevalence 13.5 9.5 7.8 5.6 13.4 12.7 7.8 5.6

*13.3 9.3- 7.6- 5.4- 13.2- 12.5- 7.6- 5.4-*

*13.7 9.7 8.0 5.8 13.6 12.9 8.0 5.8*

% on ART^+^ 8.6 6.6 5.7 4.4 8.2 7.5 5.5 4.3

*8.5- 6.5- 5.6- 4.3- 8.1- 7.4- 5.4- 4.2-*

*8.7 6.7 5.8 4.5 8.3 7.6 5.6 4.4*

death rate 1.19 1.07 1.01 0.93 1.14 1.10 0.99 0.93

*1.17- 1.04- 0.98- 0.90- 1.11- 1.07- 0.96- 0.90-*

*1.21 1.10 1.03 0.96 1.17 1.13 1.02 0.96*

% with on-going 0% 46% 46% 46% 23% 23% 46% 46%

vaccine effect  *0%- 46%- 46%- 46%- 23%- 23%- 46%- 46%-*

*0% 46% 46% 46% 23% 23% 46% 46%*

Prophylactic vaccine effect incidence 7.7 5.3 5.2 4.1 6.4 5.2 4.2 2.7

in 50% of people *7.6- 5.1- 5.0- 3.9- 6.2- 5.0- 4.0- 2.5-*

*7.8 5.5 5.4 4.3 6.6 5.4 4.4 2.9*

prevalence 13.5 11.1 10.8 9.2 12.9 12.0 10.1 8.2

*13.3 10.9- 10.6- 9.0- 12.7- 11.8- 9.9- 8.0-*

*13.7 11.3 11.0 9.4 13.1 12.2 10.3 8.4*

% on ART^+^ 8.6 7.4 7.3 6.4 7.6 6.6 6.4 5.2

*8.5- 7.3- 7.2- 6.3- 7.5- 6.5- 6.3- 5.1-*

*8.7 7.5 7.4 6.5 7.7 6.7 6.5 5.3*

death rate 1.19 1.12 1.10 1.05 1.09 1.03 1.03 0.96

*1.17- 1.09- 1.07- 1.02- 1.06- 1.00- 1.00- 0.93-*

*1.21 1.15 1.13 1.08 1.12 1.06 1.06 0.99*

% with on-going 0% 23% 23% 23% 45% 45% 45% 45%

vaccine effect  *0%- 23%- 23%- 23%- 45%- 45%- 45%- 45%-*

*0% 23% 23% 23% 45% 45% 45% 45%*

Targeted at people having incidence 7.7 4.3 3.0 1.4 6.5 5.6 2.8 1.5

condom-less sex in past *7.6- 4.1- 2.8- 1.2- 6.3- 5.4- 2.6- 1.3-*

year with new partner *7.8 4.5 3.2 1.6 6.7 5.8 3.0 1.7*

prevalence 13.5 10.2 8.6 6.4 12.9 12.2 8.5 6.5 *13.3 10.0- 8.4- 6.2- 12.7- 12.0- 8.3- 6.3-*

*13.7 10.4 8.8 6.6 13.1 12.4 8.7 6.7*

% on ART^+^ 8.6 7.0 6.1 4.8 7.8 7.0 5.8 4.8

*8.5- 6.9- 6.0- 4.7- 7.7- 6.9- 5.7- 4.7-*

*8.7 7.1 6.2 4.9 7.9 7.1 5.9 4.9*

death rate 1.19 1.09 1.03 0.96 1.12 1.06 1.01 0.95

*1.17- 1.06- 1.00- 0.93- 1.09- 1.03- 0.98- 0.92-*

*1.21 1.12 1.06 0.99 1.15 1.09 1.04 0.98*

% with on-going 0% 32% 32% 32% 32% 32% 32% 32%

vaccine effect  *0%- 32%- 32%- 32%- 32%- 32%- 32%- 32%-*

*0% 32% 32% 32% 32% 32% 32% 32%*

----------------------------------------------------------------------------------------------------------------------------------------------------------------

+ of entire population, including HIV uninfected. ** for each person given a booster there is only an 80% chance they receive the next booster.

**Figure S1.** Predicted outcomes 2025-2060 of eight vaccine introduction scenarios in 2025: (i) prevention efficacy 0.0, viral load efficacy 0.0 log_10_, (ii) prevention efficacy 0.3, viral load efficacy 0.0 log_10_, (iii) prevention efficacy 0.5, viral load efficacy 0.0 log_10_, (iv) prevention efficacy 0.9, viral load efficacy 0.0 log_10_, (v) prevention efficacy 0.0, viral load efficacy 1.0 log_10_, (vi) prevention efficacy 0.0, viral load efficacy 2.0 log_10_, (vii) prevention efficacy 0.5, viral load efficacy 1.0 log_10_, (viii) prevention efficacy 0.9, viral load efficacy 2.0 log_10_. All in the context of vaccination at 15, with a rate of vaccination per 3 months of 0.3 amongst those age 15-17 (and a 5 year catch-up program amongst adults age 18-30 covering 50% of the population of that age), with a maximum coverage (in 15-17 year olds) of 0.7, and with regular boosters every 5 years (the assumed duration of vaccine effect) with 80% of people being adherent to these boosts. See footnote for full description of variable definition.

Figure S1a

Figure S1b

Figure S1c

Figure S1d

Figure S1e

Figure S1f

Figure S1g

Figure S1h

Figure S1i

Figure S1j

Figure S1k

Figure S1l

Figure S1m

Figure S1n

Figure S1o

**Footnote: Definition of variables for Figure 2 and Figure S1**

all age 15-65 unless stated

Fig 2a. incidence of hiv

numerator: number of people developing HIV in the year

denominator: person years lived in population without hiv in the year

Fig 2b. prevalence of hiv

numerator: number of people living with HIV

denominator: whole population

Fig 2c. death rate in hiv infected population

numerator: number who died in a period

denominator: number living with HIV

Fig 2d. death rate in whole population

numerator: number who died in a period

denominator: whole populaation

Fig 2e. proportion of people on art amongst the whole population

numerator: number on art

denominator: whole population

Fig 2f. Proportion of whole population with current viral load > 1000 copies/mL

numerator: number of people with viral load > 1000 copies/mL

denominator: whole population

Fig 2g. proportion of people with hiv who have current cd4 count < 350

numerator: number with HIV with CD4 count < 350

denominator: number living with HIV

Fig 2h. proportion of people with hiv who are diagnosed

numerator: number with HIV who are diagnosed

denominator: number living with HIV

Fig 2i. proportion with any current prophylactic vaccine effect

numerator: number of HIV negative people with current vaccine efficacy (i.e. vaccinated and still within the period of some vaccine effect)

denominator: number of HIV negative people in the population

Fig S1a. proportion with current aids condition

numerator: number who are currently experiencing an AIDS disease (AIDS diseases assumed to last 3 months)

denominator: number of people living with HIV

Fig S1b. proportion with aids ever diagnosed

numerator: number with current or previous AIDS disease

denominator: number living with HIV

Fig S1c. incidence of first aids diagnosis

numerator: number who develop a first AIDS event in the year

denominator: number person years lived with HIV without previous AIDS in the year

Fig S1d. death rate (aids-related death only) in hiv infected population

numerator: number who died of an AIDS disease in a period

denominator: number living with HIV

Fig S1e. rate of art initiation in hiv infected population

numerator: number who initiated art in a period

denominator: number living with HIV

Fig S1f. rate of art initiation in the diagnosed hiv infected population

numerator: number who initiated art in a period

denominator: number living with diagnosed HIV

Fig S1g. proportion of people with hiv who are on art

numerator: number on art

denominator: number living with HIV

Fig S1h. proportion of people with diagnosed hiv who are on art

numerator: number on art

denominator: number living with diagnosed HIV

Fig S1i. proportion of people with hiv who have current cd4 count < 500

numerator: number with HIV with CD4 count < 500

denominator: number living with HIV

Fig S1j. rate of art initiation amongst people with hiv

numerator: number initiating ART in period

denominator: number living with HIV

Fig S1k. incidence of first cd4 count < 350 amongst hiv infected population

numerator: number with CD4 count < 350 for the first time in period

denominator: number living with HIV

Fig S1l. proportion of people with hiv who have current viral load < 1000

numerator: number with HIV with viral load < 1000

denominator: number living with HIV

Fig S1m. proportion of people with hiv under care

numerator: number with HIV who visit HIV clinic in the period

denominator: number living with HIV

Fig S1n. proportion of people without hiv who are vaccinated

numerator: number of HIV negative people who were ever previously given a vaccine (whether currently covered the vaccine effect or not)

denominator: number of HIV negative people in the population

Fig S1o. proportion with any current viral load vaccine effect

numerator: number of HIV negative people with current vaccine efficacy (i.e. vaccinated and still within the period of some vaccine effect)

denominator: number of HIV negative people in the population
